# ﻿Seven new “cryptic” species of Discodorididae (Mollusca, Gastropoda, Nudibranchia) from New Caledonia

**DOI:** 10.3897/zookeys.1152.98258

**Published:** 2023-03-07

**Authors:** Julie Innabi, Carla C. Stout, Ángel Valdés

**Affiliations:** 1 Department of Biological Sciences, California State Polytechnic University Pomona, 3801 West Temple Avenue, Pomona, California 91768, USA California State Polytechnic University Pomona Pomona United States of America

**Keywords:** Molecular phylogenetics, species delimitation, systematics, taxonomy

## Abstract

The study of a well-preserved collection of discodorid nudibranchs collected in Koumac, New Caledonia, revealed the presence of seven species new to science belonging to the genera *Atagema*, *Jorunna*, *Rostanga*, and *Sclerodoris*, although some of the generic assignments are tentative as the phylogeny of Discodorididae remains unresolved. Moreover, a poorly known species of *Atagema* originally described from New Caledonia is re-described and the presence of *Sclerodoristuberculata* in New Caledonia is confirmed with molecular data. All the species described herein are highly cryptic on their food source and in the context of the present study the term “cryptic” is used to denote such species. This paper highlights the importance of comprehensive collecting efforts to identify and document well-camouflaged taxa.

## ﻿Introduction

The systematics of sea slugs has benefited enormously from the introduction of molecular data analyses, which have dramatically improved species delimitation and phylogenetic reconstruction, facilitating the description and re-description of taxa belonging to notoriously difficult taxonomic groups (e.g., Jörger et al. 2012; Churchill et al. 2014; Ekimova et al. 2015; Martín‐Hervás et al. 2021). In this context, the term “cryptic” is widely used to refer to taxa that are morphologically indistinguishable but can be identified or distinguished using molecular data (Jörger and Schrödl 2013). On the contrary, in ecological research, the term “cryptic” has long been used to denote organisms that are camouflaged on their environment or food source (Faulkner and Ghiselin 1983; Dalton and Godwin 2006; Cheney et al. 2014), and this is not uncommon in many sea slug lineages. But, while “cryptic” species of sea slugs and nudibranchs in the systematics sense have received a great deal of attention in recent years, resulting in the description and identification of numerous cryptic species (e.g., Epstein et al. 2019; Knutson and Gosliner 2022), “cryptic” sea slugs in the ecological sense have been somewhat neglected and have received significantly less attention compared to their often brightly colored, extravagantly shaped cousins.

“Cryptic” species of sea slugs in the ecological sense are difficult to collect, requiring a substantial effort by experienced collectors, or the collection and processing of substrate suspected to contain living specimens. With the exception of sacoglossans, for which substrate collection produces specimens relatively easily (Krug et al. 2016, 2018), few examples of papers describing ecologically cryptic sea slug species have been published in recent years (e.g., Pola et al. 2012; Donohoo and Gosliner 2020).

In this paper we examine a few ecologically “cryptic” species of dorid nudibranchs collected during three research expeditions to Koumac, New Caledonia. These expeditions included a multidisciplinary team of expert collectors and taxonomists, using a combination of a variety of collecting techniques and methods (direct collecting, substrate collecting, autonomous reef monitoring structures (ARMS), underwater vacuum-cleaners, brush baskets, dredging, ROVs, etc.), resulting in an exceptionally well-curated collection. Among the specimens collected were several extraordinarily cryptic species in the ecological sense that would have been difficult to detect without the collecting infrastructure of the Koumac expeditions.

All the species described or re-described herein belong to the family Discodorididae. While this group has been the subject of several monographic reviews (Valdés and Gosliner 2001; Valdés 2002; Dayrat 2010) there is no consensus on the taxonomic structure of the Discodorididae or the number of valid genera. Additionally, molecular phylogenies including substantial coverage of this group (e.g., Mahguib and Valdés 2015; Hallas et al. 2017) have failed to provide enough support to unravel the relationships among different clades. In the present study we use a newly generated molecular phylogeny, including a broad representation of Discodorididae genera, as well as morphological data to provide a framework of classification for the new species described. In some cases, this information is not sufficient to provide definitive generic placements and therefore they are left as tentative.

## ﻿Materials and methods

### ﻿Source of specimens

The material examined in this study was collected during three expeditions to Koumac, New Caledonia, organized by the "Muséum national d’Histoire naturelle, Paris, France (**MNHN**). All collected specimens were individually photographed, labeled, preserved in 95% ethanol, and deposited at the MNHN. A total of 56 specimens was examined in this study, 46 of which were successfully sequenced (Table 1).

**Table 1.** List of specimens examined in this paper, including isolate, voucher and GenBank accession numbers when available. Specimens labeled with an asterisk (*) were successfully sequenced for this study.

Species	Isolate	Voucher	GenBank Accession Numbers		
			COI	16S	H3
*Aldisaalbatrossae*	JM153a	CASIZ 181288	KP871632	KP871679	KP871655
*Asteronotuscespitosus*	–	CASIZ 191163	MN720294	MN722441	MN720325
	–	CASIZ 191321	MN720296	MN722443	MN720327
*Asteronotushepaticus*	–	–	MW559976	MW559976	–
	–	CASIZ 191310	MN720295	MN722442	MN720326
*Asteronotusmarkaensis*	–	CASIZ 192316A	MN720299	MN722446	MN720330
*Asteronotusmimeticus*	–	CASIZ 208221	MN720305	MN722452	MN720336
*Asteronotusnamuro*	–	CASIZ 192297	MN720298	MN722445	MN720329
*Asteronotusspongicolus*	–	CASIZ 192317A	MN720300	MN722447	MN720331
	–	CASIZ 194597	MN720301	MN722448	MN720332
Atagemaspongiosa*	JI09	MNHN IM-2013-86190	OQ362153	OQ379356	OQ366207
	JI30	MNHN IM-2013-86189	OQ362156	–	OQ366210
	JI33	MNHN IM-2013-86188	OQ362154	–	OQ366208
*Atagemaspongiosa*	JI02	MNHN IM-2013-86170	OQ362155	OQ379357	OQ366209
Atagemacf.osseosa	–	CASIZ 185142	MF958426	MF958296	–
*Atagemanotacristata*	–	CASIZ 167980	KP871634	KP871681	KP871657
*Atagemapapillosa*	JI42	MNHN IM-2013-86192	OQ362138	–	OQ366192
*Atagemasobanovae* sp. nov.*	JI16	MNHN IM-2013-86211	OQ362139	–	OQ366193
	JI05	MNHN IM-2013-86181	OQ362141	OQ379351	OQ366195
	JI06	MNHN IM-2013-86178	OQ362140	OQ379350	OQ366194
	JI08	MNHN IM-2013-86187	OQ362142	OQ379352	OQ366196
	JI19	MNHN IM-2013-86174	OQ362150	OQ379354	OQ366204
	JI21	MNHN IM-2013-86179	OQ362145	OQ379353	OQ366199
	JI23	MNHN IM-2013-86180	OQ362151	OQ379355	OQ366205
	JI28	MNHN IM-2013-86175	OQ362146	–	OQ366200
	JI29	MNHN IM-2013-86171	OQ362148	–	OQ366202
	JI41	MNHN IM-2013-86173	OQ362149	–	OQ366203
	JI44	MNHN IM-2013-86176	OQ362147	–	OQ366201
	JI45	MNHN IM-2013-86177	OQ362143	–	OQ366197
	JI46	MNHN IM-2013-86172	OQ362144	–	OQ366198
*Atagemasobanovae* sp. nov.	–	MNHN IM-2013-86229	–	–	–
*Atagemakimberlyae* sp. nov.*	JI43	MNHN IM-2013-86191	OQ362152	–	OQ366206
*Carminodorisflammea*	–	CASIZ 177628	MN720285	MN722433	MN720311
*Diaululagreeleyi*	TL286	LACM 3016	KU950017	KU949947	KU950060
*Diaululanayarita*	TL176	LACM 153353	KU950018	KU949948	KU950061
*Diaululaodonoghuei*	TL178	CPIC 01073	KU950036	KU949967	KU950080
	TL179	CPIC 01074	KU950037	KU949968	KU950081
*Diaululasandiegensis*	TL025	CPIC 00911	KU950057	KU949987	KU950103
	TL268	CPIC 01269	KU950058	KU949989	KU950105
*Discodorisboholiensis*	–	CASIZ 204802	MN720304	MN722451	MN720335
*Discodoriscebuensis*	–	CASIZ 185141	KP871639	KP871687	KP871663
	–	CASIZ 190761	MN720293	MN722440	MN720322
*Discodoriscoerulescens*	–	CASIZ 182850	MF958421	MF958290	–
*Doriskerguelenensis*	H20	–	EU823146	EU823238	–
*Dorispseudoargus*	–	–	AJ223256	AJ225180	–
*Hexabranchussanguineus*	JM70a	CPIC 00336	KP871644	KP871692	KP871668
*Hoplodorisdesmoparypha*	–	CASIZ 070066	MN720283	MN722431	MN720309
	–	CASIZ 309550	MN720308	MN722455	–
*Hoplodorisrosans*	–	CASIZ 182837	MN720288	MN722436	MN720318
	–	CASIZ 182921	MN720290	MN722438	MN720320
*Jorunnaartsdatabankia*	–	NTNU-VM 58891	MW784174	MW784486	MW810589
	–	ZMBN 125946	MW784173	MW784485	MW810590
	–	ZMBN 127749	MW784172	MW784487	–
*Jorunnadaoulasi* sp. nov.	–	MNHN IM-2013-86230	–	–	–
	–	MNHN IM-2013-86220	–	–	–
*Jorunnadaoulasi* sp. nov.*	JI22	MNHN IM-2013-86219	OQ362165	OQ379361	OQ366219
*Jorunnahervei* sp. nov.	–	MNHN IM-2013-86221	–	–	–
	–	MNHN IM-2013-86222	–	–	–
	–	MNHN IM-2013-86223	–	–	–
	–	MNHN IM-2013-86224	–	–	–
*Jorunnahervei* sp. nov.*	JI47	MNHN IM-2013-86225	OQ362163	–	OQ366217
	JI48	MNHN IM-2013-86226	OQ362164	–	OQ366218
*Jorunnahervei* sp. nov.	–	MNHN IM-2013-86227	–	–	–
	–	MNHN IM-2013-86228	–	–	–
*Jorunnaliviae*	–	MNCN15.05/200187	OP948382	–	–
	–	MNCN15.05/200188	OP948383	–	–
	–	MNCN15.05/200189	OP948384	–	–
	–	MNCN15.05/94693	OP948385	–	–
*Jorunnaonubensis*	–	ZMBN 125474	MW784171	MW784483	MW810587
*Jorunnatomentosa*	–	CASIZ 175752	MW784185	MW784508	MW810604
	–	CASIZ 175753	MW784202	MW784506	MW810610
	–	CASIZ 176820	MW784179	–	MW810602
	–	CASIZ 193035	MW784176	MW784491	MW810607
*Montereinanobilis*	–	CASIZ 182223	HM162684	HM162593	HM162499
*Paradorisliturata*	–	CASIZ 177510	KP871648	KP871696	–
	–	CASIZ 182756	MW223084	MW220951	MW415015
*Peltodorisatromaculata*	–	–	AF249784	AF430360	–
*Platydorissanguinea*	–	CASIZ 177762	MF958416	MF958285	–
*Rostangabyga*	–	CASIZ 181157	MW223085	MW220952	MW415016
*Rostangacalumus*	EED-Phy-934	–	FJ917485	FJ917427	–
*Rostangaelandsia*	–	CASIZ 176110	KP871651	KP871699	KP871674
*Rostangapoddubetskaiae* sp. nov.*	JI01	MNHN IM-2013-86199	OQ362134	OQ379347	OQ366188
	JI03	MNHN IM-2013-86202	OQ362129	–	OQ366183
	JI07	MNHN IM-2013-86218	OQ362136	OQ379348	OQ366190
	JI12	MNHN IM-2013-86203	OQ362122	OQ379345	OQ366176
	JI13	MNHN IM-2013-86206	OQ362121	–	OQ366175
	JI15	MNHN IM-2013-86215	OQ362124	–	OQ366178
	JI17	MNHN IM-2013-86200	OQ362125	–	OQ366179
	JI18	MNHN IM-2013-86209	OQ362135	–	OQ366189
	JI20	MNHN IM-2013-86204	OQ362137	OQ379349	OQ366191
	JI24	MNHN IM-2013-86216	OQ362127	OQ379346	OQ366181
	JI25	MNHN IM-2013-86208	OQ362119	OQ379344	OQ366173
	JI26	–	OQ362132	–	OQ366186
	JI27	MNHN IM-2013-86205	OQ362130	–	OQ366184
	JI31	MNHN IM-2013-86212	OQ362126	–	OQ366180
	JI32	MNHN IM-2013-86201	OQ362133	–	OQ366187
	JI36	MNHN IM-2013-86213	OQ362120	–	OQ366174
	JI37	MNHN IM-2013-86214	OQ362123	–	OQ366177
	JI38	MNHN IM-2013-86217	OQ362128	–	OQ366182
	JI39	MNHN IM-2013-86207	OQ362131	–	OQ366185
*Rostangapoddubetskaiae* sp. nov.	JI40	MNHN IM-2013-86210	–	–	–
*Rostangapulchra*	–	CASIZ 174490A	MW223086	MW220953	MW415017
*Sclerodoris* sp.	–	CASIZ 182866	MN720289	MN722437	MN720319
	–	CASIZ 191525	MN720297	MN722444	MN720328
*Sclerodorisfaninozi* sp. nov.*	JI11	MNHN IM-2013-86198	OQ362161	OQ379359	OQ366215
*Sclerodorisdutertrei* sp. nov.*	JI04	MNHN IM-2013-86193	OQ362157	OQ379358	OQ366211
	JI14	MNHN IM-2013-86196	OQ362160	–	OQ366214
	JI34	MNHN IM-2013-86195	OQ362159	–	OQ366213
	JI35	MNHN IM-2013-86194	OQ362158	–	OQ366212
*Sclerodoristuberculata*	–	CASIZ 190788	MF958417	MF958286	MN720323
Sclerodoristuberculata*	JI10	MNHN IM-2013-86197	OQ362162	OQ379360	OQ366216
*Taringa* sp.	–	CASIZ 172039	MN720284	MN722432	MN720310
*Taringatelopia*	–	CASIZ 182933	MN720291	KP871700	KP871675
*Tayuvaketos*	TL086	CPIC 00654	KU950019	KU949949	KU950062
Thordisaaff.albomacula	–	CASIZ 179590	MF958418	MF958287	MN720313
	–	CASIZ 181136	MN720286	MN722434	MN720314
	–	CASIZ 182834	MT454622	MT452888	MT454628
	–	CASIZ 220322	MT454620	MT452884	MT454624
*Thordisabimaculata*	–	CASIZ 184516	MN720292	MN722439	MN720321
*Thordisanieseni*	–	CASIZ 173057	MW223087	MW220954	MW415018

### ﻿DNA extraction, amplification, and sequencing

From each specimen a small tissue sample (~ 1 mm^3^) was taken from the foot using sterilized forceps. DNA extraction was conducted using a Chelex protocol using a mixture of 200 µL of 10% Chelex 100 (Bio-Rad.com), blotted tissue (to remove any remaining ethanol), and 4 µL of proteinase K. The 1.7 mL microcentrifuge tubes with the mixture were placed in a water bath for 20 min at 55 °C (cell lysis and protein digestion) followed by placement in a heat block at 100 °C for 8 min (protein denaturation). Then, the microcentrifuge tubes were centrifuged to separate the Chelex beads from the supernatant containing the DNA, and 100 µL of the supernatant was aliquoted and used for DNA amplification.

The Polymerase Chain Reaction (PCR) was conducted on all samples for three genes: cytochrome c oxidase subunit one (CO1, mtDNA), ribosomal RNA 16S (16S, mtDNA), and Histone H3 (H3, nuclear), using universal primers (Folmer et al. 1994; Palumbi 1996; Colgan et al. 1998) in a Thermal Cycler T100 (Thermo Scientific, Waltham, MA). Each reaction was conducted using 38.5 µL of ultra-pure water, 5 µL of 10× PCR Dream Taq Buffer, 1.25 µL of Bovine Serum Albumin (BSA 20mg/mL), 1 µL 10 mM dNTPS, 1 µL forward primer, 1 µL reverse primer, 0.25 µL of Dream Taq, and 2 µL of DNA extraction, which resulted in each microcentrifuge tube containing a total volume of 50 µL. Reaction conditions for 16S and H3 were are follows: initial denaturation at 94 °C for 2 min, denaturation at 94 °C for 30 sec, annealing at 50 °C for 30 sec, elongation at 68 °C for 1 min, 30 cycles from denaturation to elongation and a final elongation at 68 °C for 7 min. Reaction conditions for COI were are follows: initial denaturation at 95 °C for 3 min, denaturation at 94 °C for 45 sec, annealing at 45 °C for 45 sec, elongation at 72 °C for 2 min, 35 cycles from denaturation to elongation and a final elongation at 72 °C for 10 min. Gel electrophoresis was conducted using 1% agarose tris-borate-EDTA (TBE) buffer and ethidium bromide for 15 min, including a ladder and a negative control to verify successful amplification of the PCR products of the correct length and confirm the absence of contamination. DNA purification was conducted with E.Z.N.A Cycle Pure D6492-02 kits (Omega Bio-Tek, Inc., Norcross, GA) following the manufacturer’s instructions. DNA concentration of purified samples was measured using a Nano Drop 1000 spectrophotometer (Thermo Scientific, Waltham, MA) prior to Sanger sequencing, which was outsourced to Retrogen Inc. (San Diego, CA).

### ﻿Data analysis

Forward and reverse sequences were assembled, edited, and consensus sequences were extracted using the computer program Geneious v. 11.1.5 (Kearse et al. 2012). Additional sequences were downloaded from GenBank for comparison (Table 1). Sequences were aligned using the MUSCLE (Edgar 2004) plug-in in Geneious. Gaps in the 16S alignment were removed manually, and concatenation of all three genes was performed in Geneious. Bayesian and maximum likelihood phylogenetic analyses were conducted on the concatenated sequences (partitioned by gene) and on each gene fragment individually. Bayesian analysis was implemented using MrBayes v. 3.2.1 (Ronquist et al. 2012) with the GTR model, using two runs of six chains for 10 million repetitions with a sampling interval of 1,000 repetitions and burn-in of 25% removed. The maximum-likelihood analysis was conducted in RaXMLGUI v. 1.0 (Silvestro and Michalak 2012) using the bootstrap + consensus option and the GAMMAGI model with 10,000 bootstrap repetitions. *Hexabranchussanguineus* (Rüppell & Leuckart, 1830) was used to root the resulting trees. Nodes in the resulting phylogenetic tree with Posterior probabilities (PP) ≥ 90% and bootstraps values (MLB) ≥ 70% were interpreted as supported.

The Automatic Barcode Gap Discovery (ABGD) software (Puillandre et al. 2012) was used to provide statistical support to determine the number of species in the sample using COI sequences of 107 specimens. Pairwise p-distance values were calculated using MEGA v. 11.0.13 (Kumar et al. 2018) using the Kimura-2 model (Kimura 1980).

### ﻿Morphological examination

At least two specimens (if available) from each species recovered in the ABGD analysis were dissected to study their reproductive system (including the penis), jaw (if present), and radula. Dissections were performed by a dorsal incision from the middle of the nudibranch to the anterior end. The reproductive system was carefully removed from each specimen and drawn with a camera lucida. The penis was dissected and examined under a compound microscope. The buccal mass (including the radula and jaw) was removed from the anterior end of each animal and placed in a 10% NaOH solution to dissolve soft tissue and expose the radula and jaws. After 20 min to several hours, the radula and labial cuticle (housing the jaw) were rinsed in distilled water and mounted on a small copper plate for Scanning Electron Microscopy (SEM) examination. The samples were sputter-coated with gold and observed under a JSM- 6010PLUS/LA SEM at California Polytechnic State University, Pomona, California.

## ﻿Results

**Figure 1.**
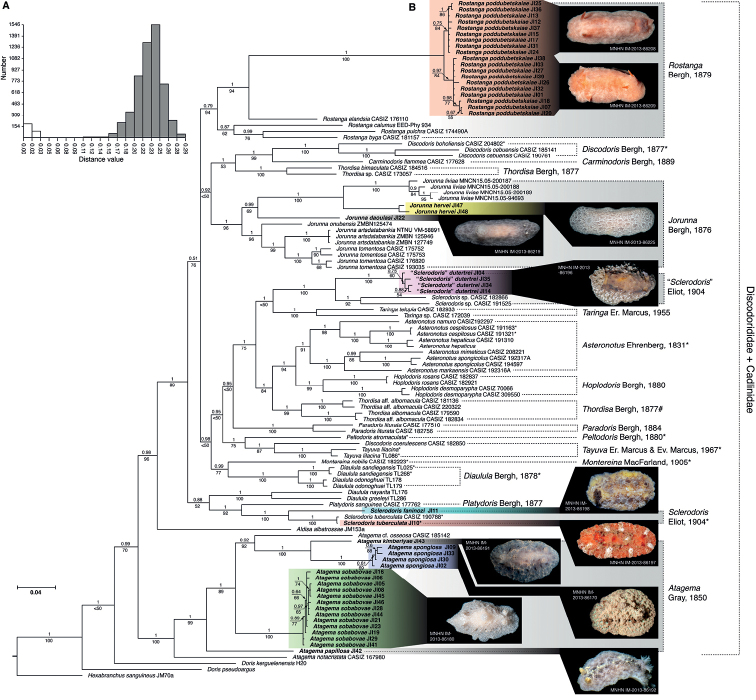
Graphic representation of the molecular analyses results **A** histogram represents the distance plot for the ABGD analysis using the COI gene showing pairwise *p*-distances (Kimura 2 model) among candidate species **B** Bayesian consensus tree of the concatenated 16S, COI and H3 gene fragments. Posterior probabilities from the Bayesian analysis are listed above each branch; bootstrap values from maximum likelihood analysis are listed below each branch.

The concatenated phylogenetic trees (BI and ML) recovered species of Discodorididae Bergh, 1891 + Cadlinidae Bergh, 1891 (represented by the genus *Aldisa* Bergh, 1878) as a monophyletic group (PP = 0.99, MLB = 70) (Fig. 1). Members of the genus *Atagema* Gray, 1850 are monophyletic (PP = 1, MLB = 100) and sister to the rest of Discodorididae + Cadlinidae (PP = 0.98, MLB = 96). The remaining Discodorididae (when *Aldisa* and *Atagema* are excluded) is monophyletic (PP = 1, MLB = 89) and contains a number of clades, most of which are not supported. The analyses recovered a clade containing species identified as *Sclerodoris* Eliot, 1904 (including the type species, *S.tuberculata* Eliot, 1904), as monophyletic (PP = 1, MLB = 100), which is sister to the single representative of *Platydoris* Bergh, 1877 (PP = 1, MLB = 92); these two groups appear to be related to another monophyletic group (PP = 1, MLB = 100), containing two species identified as members of *Diaulula* Bergh, 1878 [*D.nayarita* (Ortea & Llera, 1981) and *D.greeleyi* (MacFarland, 1909)], but the relationship between *Platydoris*, *Sclerodoris*, and these two species of *Diaulula* is not supported. Another monophyletic group includes species identified as *Rostanga* Bergh, 1879 (PP = 0.79, MLB = 94), with unresolved relationships to other Discodorididae clades. The genus *Discodoris* Bergh, 1877, including the type species *D.boholiensis* Bergh, 1877, is also monophyletic (PP = 1, MLB = 100) and sister to the single representative of *Carminodoris* Bergh, 1889 (PP = 1, MLB = 99), and these two groups appear to be related to some species identified as *Thordisa* Bergh, 1877, which also form a monophyletic group (PP = 1, MLB = 100). Although not supported in the ML analysis, *Discodoris*, *Carminodoris*, and *Thordisa* appear to be related to the monophyletic genus *Jorunna* Bergh, 1876 (PP = 1, MLB = 96), including the type species *J.tomentosa* (Cuvier, 1804). Another genus recovered as monophyletic is *Asteronotus* Ehrenberg, 1831 (PP = 1, MLB = 91), including the type species *A.cespitosus* (van Hasselt, 1824), which is sister (PP = 1, MLB = 94) to another monophyletic group including species identified as *Hoplodoris* Bergh, 1880 (PP = 1, MLB = 99), and together sister (PP = 1, MLB = 75) to another group of species identified as *Thordisa* (PP = 1, MLB = 99). Other traditional genus-level groups appear to be related but these relationships are not supported; these include *Paradorisliturata* (Bergh, 1905), the type species of *Peltodoris* Bergh, 1880 (*P.atromaculata* Bergh, 1880), *Discodoriscoerulescens* Bergh, 1888, and specimens currently identified as *Tayuvalilacina* (Gould, 1852). Finally, the genus *Diaulula* including the type species *D.sandiegensis* (Cooper, 1863) is monophyletic (PP = 1, MLB = 100) and sister to *Montereinanobilis* MacFarland, 1905. Single gene fragment phylogenetic analyses provided similar results in general but with lower resolution (Suppl. materials 1–3).

The ABGD analysis recovered 52 distinct species in the sample, which matches the structure recovered in the phylogenetic analysis (Table 2). The species recovered include all the taxa described below in the systematics section and species currently recognized as valid in the literature. The only exceptions are *Diaululasandiegensis* and *Diaululaodonohuei* (Steinberg, 1963), which ABGD failed to recover as distinct, and specimens identified as *Discodoriscebuensis* Bergh, 1877, which ABGD recovered as two distinct species.

**Table 2.** Candidate species (groups) recovered in the ABDG analysis of COI sequence fragments. Initial Partition with prior maximal distance *P* = 2.15e^-02^; barcode gap distance = 0.088; distance simple distance minimum slope = 1.00.

Group	Species	Voucher # (Isolate #)
1	*Aldisaalbatrossae*	CASIZ 181288 (JM153a)
2	*Asteronotuscespitosus*	CASIZ 191321, CASIZ 191163
3	*Asteronotushepaticus*	n/a, CASIZ 191310
4	*Asteronotusmarkaensis*	CASIZ 192316A
5	*Asteronotusmimeticus*	CASIZ 208221
6	*Asteronotusnamuro*	CASIZ 192297
7	*Asteronotusspongicolus*	CASIZ 192317A, CASIZ 194597
8	Atagemacf.osseosa	CASIZ 185142
9	*Atagemanotacristata*	CASIZ 167980
10	*Atagemakimberlyae* sp. nov.	MNHN IM-2013-86191 (JI43)
11	*Atagemapapillosa*	MNHN IM-2013-86192 (JI42)
12	*Atagemasobanovae* sp. nov.	MNHN IM-2013-86211 (JI16), MNHN IM-2013-86178 (JI06), MNHN IM-2013-86181 (JI05), MNHN IM-2013-86187 (JI08), MNHN IM-2013-86177 (JI45), MNHN IM-2013-86172 (JI46), MNHN IM-2013-86179 (JI21), MNHN IM-2013-86175 (JI28), MNHN IM-2013-86176 (JI44), MNHN IM-2013-86171 (JI29), MNHN IM-2013-86173 (JI41), MNHN IM-2013-86174 (JI19), MNHN IM-2013-86180 (JI23)
13	*Atagemaspongiosa*	MNHN IM-2013-86190 (JI09), MNHN IM-2013-86188 (JI33), MNHN IM-2013-86170 (JI02), MNHN IM-2013-86189 (JI30)
14	*Carminodorisflammea*	CASIZ 177628
15	*Diaululagreeleyi*	LACM 3016 (TL286)
16	*Diaululanayarita*	LACM 153353 (TL176)
17	*Diaululasandiegensis/odonoghuei*	CPIC 00911 (TL025), CPIC 01269 (TL268), CPIC 01073 (TL178), CPIC 01074 (TL179)
18	*Discodorisboholiensis*	CASIZ 204802
19	*Discodoriscebuensis*	CASIZ 185141
20	*Discodoriscebuensis*	CASIZ 190761
21	*Discodoriscoerulescens*	CASIZ 182850
22	*Doriskerguelenensis*	(H20)
23	*Dorispseudoargus*	n/a
24	*Hexabranchussanguineus*	CPIC 00336 (JM70a)
25	*Hoplodorisdesmoparypha*	CASIZ 070066, CASIZ 309550
26	*Hoplodorisrosans*	CASIZ 182837, CASIZ 182921
27	*Jorunnaartsdatabankia*	NTNU-VM 58891, ZMBN 125946, ZMBN 127749
28	*Jorunnadaoulasi* sp. nov.	MNHN IM-2013-86219 (JI22)
29	*Jorunnahervei* sp. nov.	MNHN IM-2013-86225 (JI47), MNHN IM-2013-86226 (JI48)
30	*Jorunnaliviae*	MNCN15.05/200187, MNCN15.05/200188, MNCN15.05/200189, MNCN15.05/94693
31	*Jorunnaonubensis*	ZMBN 125474
32	*Jorunnatomentosa*	CASIZ 175752, CASIZ 175753, CASIZ 176820, CASIZ 193035
33	*Paradorisliturata*	CASIZ 177510, CASIZ 182756
34	*Peltodorisatromaculata*	n/a
35	*Montereinanobilis*	CASIZ 182223
36	*Platydorissanguinea*	CASIZ 177762
37	*Rostangabyga*	CASIZ 181157
38	*Rostangacalumus*	EED-Phy-934
39	*Rostangaelandsia*	CASIZ 176110
40	*Rostangapoddubetskaiae* sp. nov.	MNHN IM-2013-86208 (JI25), MNHN IM-2013-86213 (JI36), MNHN IM-2013-86206 (JI13), MNHN IM-2013-86203 (JI12), MNHN IM-2013-86214 (JI37), MNHN IM-2013-86215 (JI15), MNHN IM-2013-86200 (JI17), MNHN IM-2013-86212 (JI31), MNHN IM-2013-86216 (JI24), MNHN IM-2013-86209 (JI18), MNHN IM-2013-86218 (JI07), JI20 (MNHN IM-2013-86204), MNHN IM-2013-86217 (JI38), MNHN IM-2013-86202 (JI03), MNHN IM-2013-86205 (JI27), MNHN IM-2013-86207 (JI39), MNHN IM-2013-86201 (JI32), MNHN IM-2013-86199 (JI01)
41	*Rostangapulchra*	CASIZ 174490A
42	*Sclerodorisdutertrei* sp. nov.	MNHN IM-2013-86193 (JI04), MNHN IM-2013-861924 (JI35), MNHN IM-2013-86195 (JI34), MNHN IM-2013-86196 (JI14)
43	*Sclerodoris* sp.	CASIZ 182866
44	*Sclerodoris* sp.	CASIZ 191525
45	*Sclerodorisfaninozi* sp. nov.	MNHN IM-2013-86198 (JI11)
46	*Sclerodoristuberculata*	CASIZ 190788, MNHN IM-2013-86197 (JI10)
47	*Taringa* sp.	CASIZ 172039
48	*Taringatelopia*	CASIZ 182933
49	*Tayuvaketos*	n/a, CPIC 00654 (TL086)
50	Thordisaaff.albomacula	CASIZ 181136, CASIZ 220322
51	*Thordisaalbomacula*	CASIZ 179590, CASIZ 182834
52	*Thordisabimaculata*	CASIZ 184516
53	*Thordisanieseni*	CASIZ 173057

There are consistent interspecific morphological differences among representative specimens in the clades recovered in the phylogenetic analyses, which also correspond to the species from the species delimitation analyses. These differences included aspects of internal morphology such as radular morphology and reproductive system differences that are discussed in the Systematics section below.

## ﻿Systematics

### ﻿Family Discodorididae Bergh, 1891

#### ﻿Genus Atagema

Taxon classificationAnimaliaNudibranchiaDiscodorididae

Gray, 1850

9CE7F0AB-81D1-548C-9A39-44C2C0600EC8

Atagema Gray 1842–50 [1850]: 104. Type species: Doriscarinata Quoy & Gaimard, 1832 [= Atagemacarinata (Quoy & Gaimard, 1832)], by monotypy.
Trippa Bergh 1877: 63. Type species: Trippaornata Bergh, 1877 [= Atagemaornata Ehrenberg, 1831], by original designation.
Phlegmodoris Bergh 1878: 593. Type species: Phlegmodorismephitica Bergh, 1878 [= Atagemaspongiosa (Kelaart, 1858)], by subsequent designation by Valdés and Gosliner (2001).
Petelodoris Bergh, 1881: 227–228. Type species: Petelodoristriphylla Bergh, 1881 [?= Atagemaornata (Ehrenberg, 1831)], by monotypy.
Glossodoridiformia O’Donoghue, 1927: 87–89. Type species Glossodoridiformiaalba O’Donoghue, 1927 [= Atagemaalba O’Donoghue, 1927], by original designation.

##### Remarks.

For an in-depth discussion of the characteristics of the genus *Atagema* and its synonyms see Valdés and Gosliner (2001).

#### ﻿ Atagema spongiosa

Taxon classificationAnimaliaNudibranchiaDiscodorididae

(Kelaart, 1858)

AFCA7441-49C2-5189-830C-E02CE0953447

**Figure 2.**
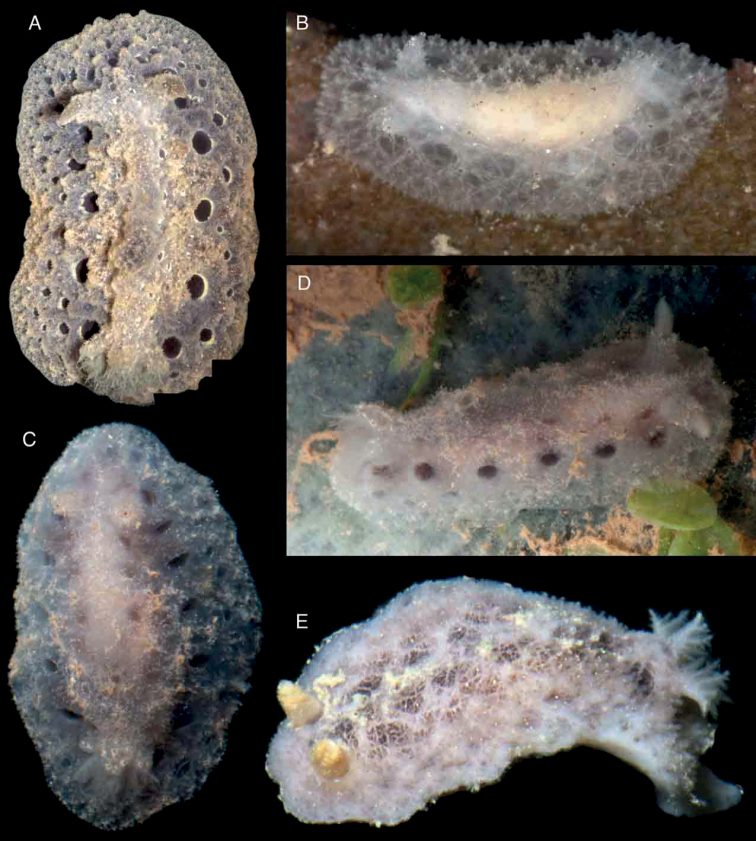
Photographs of live animals of the genus *Atagema* Gray, 1850 **A, B***Atagemaspongiosa* (Kelaart, 1858), MNHN IM-2013-86188 on black background (**A**), MNHN IM-2013-86189 in situ (**B**) **C, D***Atagemakimberlyae* sp. nov., MNHN IM-2013-86191 on black background (**C**), MNHN IM-2013-86191 in situ (**D**) **E***Atagemapapillosa* (Risbec, 1928), MNHN IM-2013-86192 on black background.

**Figure 3.**
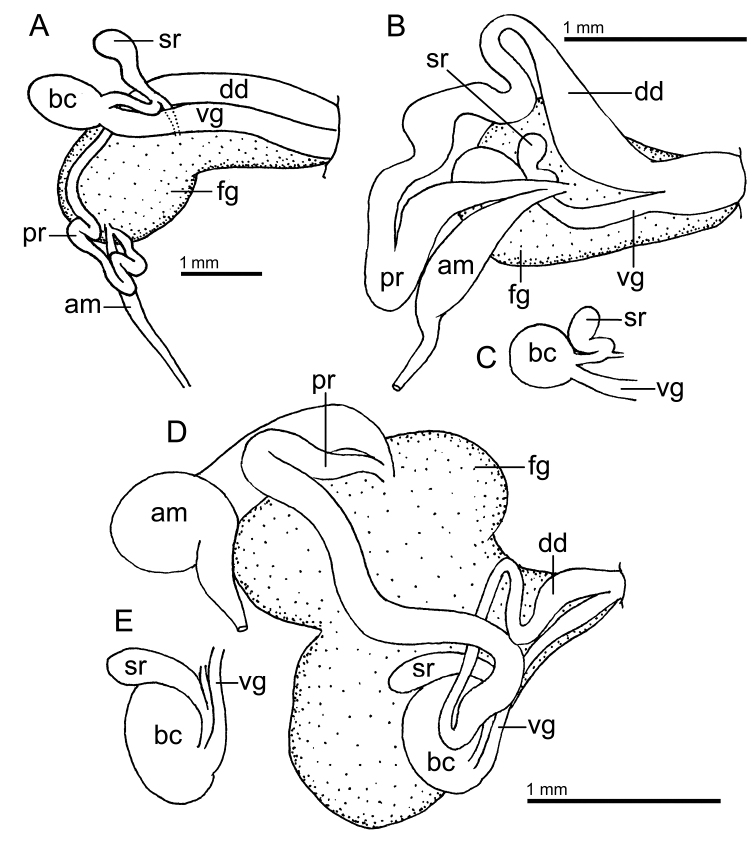
Drawings of the reproductive systems of specimens of the genus *Atagema* Gray, 1850 **A***Atagemaspongiosa* (Kelaart, 1858), MNHN IM-2013-86190 **B, C***Atagemakimberlyae* sp. nov., MNHN IM-2013-86191, general view (**B**), detail of the bursa copulatrix and seminal receptable (**C**) **D, E***Atagemapapillosa* (Risbec, 1928), MNHN IM-2013-86192, general view (**D**), detail of the bursa copulatrix and seminal receptable (**E**). Abbreviations: am, ampulla; bc, bursa copulatrix; dd, deferent duct; fg, female gland complex; pr, prostate; sr, seminal receptacle; vg, vagina.

**Figure 4.**
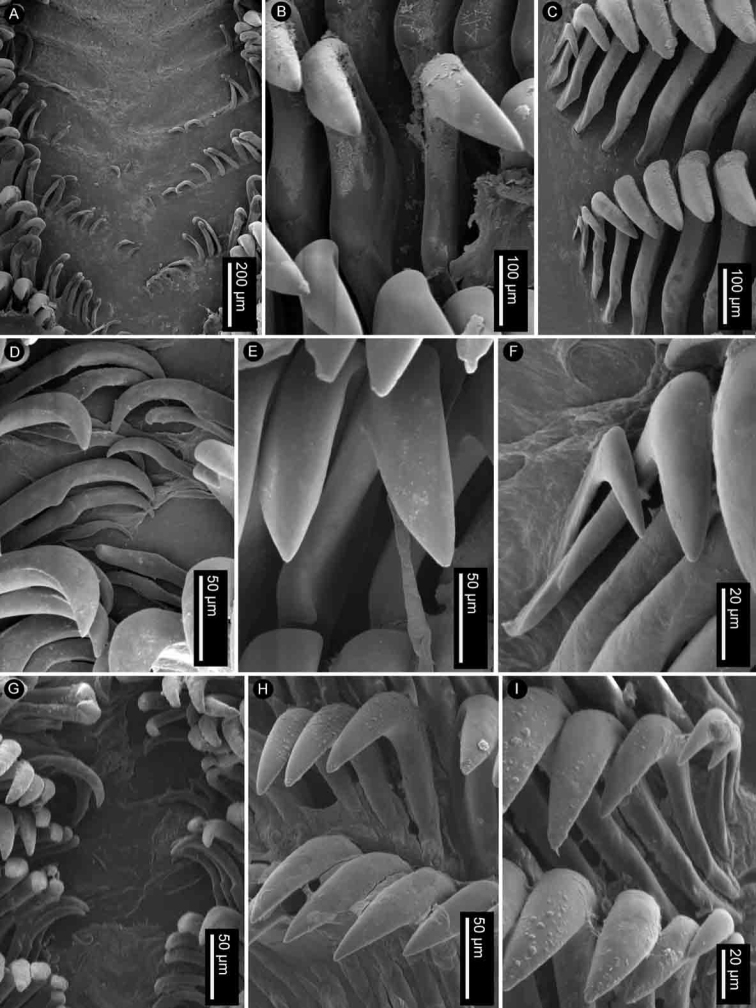
SEM of the radula of specimens of the genus *Atagema* Gray, 1850 **A–C***Atagemaspongiosa* (Kelaart, 1858), MNHN IM-2013-86190, innermost teeth (**A**), mid-lateral teeth (**B**), outer lateral teeth (**C**) **D–F***Atagemakimberlyae* sp. nov., MNHN IM-2013-86191, innermost teeth (**D**), mid-lateral teeth (**E**), outer lateral teeth (**F**) **G–I***Atagemapapillosa* (Risbec, 1928), MNHN IM-2013-86192, innermost teeth (**G**), mid-lateral teeth (**H**), outer lateral teeth (**I**).

Figs 2A, B , 3A , 4A–C

Doris spongiosa Kelaart, 1858: 97–98. Type locality: Inner Harbor, Trincomalie, Ceylon [= Trincomalee, Sri Lanka].
Doris areolata Alder & Hancock, 1864: 119, pl. 30, figs 1–3 [non Dorisareolata Stuwitz, 1835]. Type locality: Waltair, Madras Presidency [= Visakhapatnam, Andhra Pradesh], India.
Phlegmodoris mephitica Bergh, 1878: 594–597, pl. 66, figs 8–20. Type locality: Lapinig Island, Ubay, Philippines.
Trippa (Phlegmodoris) paagoumenei Risbec, 1928: 87–90, text fig. 15, pl. B, fig. 3, pl. 3, fig. 1. Type locality: Paagoumene, New Caledonia.

##### Material examined.

Pointe Pandop, Koumac, New Caledonia (20°34.9'S, 164°16.6'E), 0 m depth [Koumac 2.1 stn. KM100, rocky shore, rubble, sand, mud, seagrasses], 12 Sep 2018, 1 specimen 49 mm long (MNHN IM-2013-86170, isolate JI02). Koumac, New Caledonia (20°34.7'S, 164°16.5'E), 2–4 m depth [Koumac 2.1 stn. KR231, rocky bottom turning to mud, sponges, *Halimeda*], 25 Sep 2018, 1 specimen 27 mm long, dissected (MNHN IM-2013-86190, isolate JI09). Pointe Pandop, Koumac, New Caledonia (20°34.9'S, 164°16.6'E), 0 m depth [Koumac 2.2 stn. KM100, rocky shore, rubble, sand, mud, seagrasses], 1 Mar 2019, 1 specimen 29 mm long (MNHN IM-2013-86188, isolate JI33). Koumac, New Caledonia (20°35.1'S, 164°16.3'E), 7–8 m depth [Koumac 2.2 stn. KR231], 1 Mar 2019, 1 specimen 6 mm long (MNHN IM-2013-86189, isolate JI30).

##### Description.

Body oval, flattened, covered with large, rounded tubercles decreasing in size towards the mantle margin (Fig. 2A, B). A central, longitudinal ridge runs between the rhinophores and gill. A series of depressions on each side of the central ridge, generally decreasing in size towards the mantle margin. Entire dorsal surface, except for the depressions, covered with caryophyllidia. Branchial sheath composed of three large lobes; gill composed of five tripinnate branchial leaves, arranged horizontally in the living animal. Rhinophoral sheaths elevated; rhinophores long, lamellated, with 24 lamellae. Juvenile specimens with less marked dorsal tubercles (Fig. 2B). Body color opaque greyish brown in adult specimens, except for the depressions, which are dark brown to black (Fig. 2A); juveniles translucent gray (Fig. 2B). Rhinophores and branchial leaves are the same color as the dorsum.

Reproductive system (Fig. 3A) with a long, narrow, simple ampulla that connects with the female gland complex and an elongated, convoluted prostate, with several folds. Prostate ~ 3× as long as the ampulla. The prostate narrows slightly before expanding into the long, simple, wide deferent duct. Deferent duct several times as wide as the prostate, but shorter in length. The penis is unarmed. The vagina is long and wide, as wide as the deferent duct, and connects directly to the small, oval bursa copulatrix. The small elongate seminal receptacle also connects to the bursa copulatrix next to the vaginal connection and the short uterine duct that enters the female gland complex. The bursa copulatrix is ~ 2× as large as the seminal receptacle.

Radular formula 18 × 35.0.35 in a 27-mm long specimen (MNHN IM-2013-86190). Rachidian teeth absent. Inner and mid-lateral teeth hamate, having a small cusp and lacking denticles (Fig. 4A, B). Innermost teeth very small in comparison to mid-laterals (Fig. 4A). The teeth increase in size suddenly towards the medial portion of the half-row (Fig. 4A). Outermost teeth small, decreasing in size gradually, and hamate (Fig. 4C). No jaw was observed, labial cuticle smooth.

##### Biology.

Geographic range including the Indian and Western Pacific oceans (see synonymy and remarks). In New Caledonia it is found under rocks during the day in shallow water, from 0–8 m depth. The specimens examined were obtained by direct collection during low tide and/or SCUBA diving; they were highly cryptic on rocks covered with sponges and other encrusting organisms.

##### Remarks.

*Dorisspongiosa* Kelaart, 1858 was originally described from Sri Lanka and re-described by Valdés and Gosliner (2001), who transferred it to the genus *Atagema*, and recognized two synonyms, *Dorisareolata* Alder & Hancock, 1864 and *Phlegmodorismephitica* Bergh, 1878. This species is common across the tropical Indo-Pacific region and is well characterized and illustrated in modern literature (Wells and Bryce 1993; Yonow 2008; Hervé 2010; Gosliner et al. 2018; Nakano 2018). The specimens here examined from New Caledonia match the original description as well as the common usage of the name in the references above (see Hervé 2010).

Trippa (Phlegmodoris) paagoumenei Risbec, 1928 was originally described based on a single specimen collected in Paagoumene, northern New Caledonia, but it was later reported from Nouméa, southern New Caledonia (Risbec 1930, 1953). Risbec (1928) described *T.paagoumenei* as having a rather tough notum, dark violet in color, except towards the edges of the foot and the mantle, where it has a yellowish tint, and completely covered with purplish green, irregular tubercles. One of the specimens from Nouméa was unusual as it was covered by a bright green deposit of metallic appearance (Risbec 1953). Rudman (2002) considered *T.paagoumenei* a member of the genus *Atagema* and a synonym of *A.spongiosa*, and we concur with this opinion.

*Atagemaspongiosa* is clearly distinct from other species of *Atagema* recognized as valid in the modern literature, such as *Atagemaornata* (Ehrenberg, 1831) [= *Atagemaintecta* Kelaart, 1858] and *Atagemacarinata* (Quoy & Gaimard, 1832), illustrated and/or redescribed in Willan and Coleman (1984), Valdés and Gosliner (2001), and Rudman (2005), as well as *Atagemaechinata* (Pease, 1860), illustrated by Tibiriçá et al. (2017) and Gosliner et al. (2018). None of these species possesses the characteristic dorsal pattern of tubercles, depressions with a central ridge present in *A.spongiosa*. *Atagemaboucheti* Valdés & Gosliner, 2001, described based in a preserved specimen from New Caledonia deep water (405–411 m depth), is characterized by having the dorsum covered by large, irregularly scattered tubercles, not aligned to form ridges. Although the live color of this species is unknown, the external morphology is clearly different from other species of *Atagema* including *Atagemaspongiosa* (see Valdés and Gosliner 2001).

#### ﻿ Atagema papillosa

Taxon classificationAnimaliaNudibranchiaDiscodorididae

(Risbec, 1928)

A3FAA312-AE33-592B-ADC2-9F4D2D29DF35

Figs 2E , 3D, E , 4G–I

**Figure 5.**
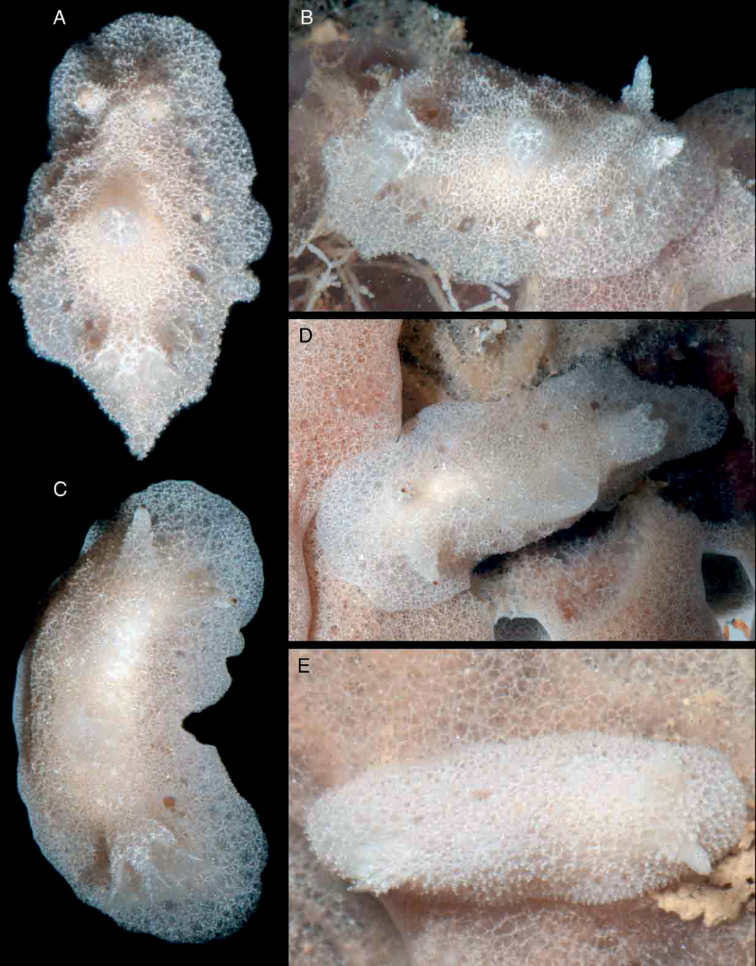
Photographs of live animals of *Atagemasobanovae* sp. nov. **A, B**MNHN IM-2013-86180, on black background (**A**), in situ (**B**) **C, D** holotype (MNHN IM-2013-86211), on black background (**A**), in situ (**B**) **E**MNHN IM-2013-86229 juvenile specimen on black background.

Phlegmodoris papillosa Risbec, 1928: 90–91, pl. 8, fig. 2. Type locality: Nouméa, New Caledonia [not indicated in the original description], see Risbec (1953).
?Trippaalbata Burn, 1962a: 101–102, text fig. 5. Type locality: Sunderland Bay, Phillip Island, Victoria, Australia.
?Atagema sp. 11: Gosliner et al. 2018: 116.

##### Material examined.

Koumac, New Caledonia (20°35.6'S, 164°16.2'E), 4–6 m depth [Koumac 2.3 stn. KD510, coral debris and coarse sand], 30 Oct 2019, 1 specimen 11 mm long, dissected (MNHN IM-2013-86192, isolate JI42).

##### Description.

Body oval, flattened, covered with a complex network of small ridges with two levels of organization (Fig. 2E). The largest ridges cover the entire body, leaving some depressions in between. Smaller ridges occur in the depressions dividing them into smaller fragments. Entire dorsal surface, except for the depressions, covered with caryophyllidia. Branchial sheath composed of three large lobes; gill composed of five tripinnate branchial leaves, arranged horizontally in the living animal. Rhinophoral sheaths elevated; rhinophores long, lamellated, with 16 lamellae. Body color opaque grey with scattered yellow spots; depressions with gray ridges dividing dark grey to black fragments. Gill leaves are the same color as the dorsum. Rhinophores greyish to yellowish cream.

Reproductive system (Fig. 3D, E) with a large, folded ampulla that connects with the female gland complex and an elongate prostate. The prostate is much longer and ~ 2× as narrow as the ampulla. The prostate narrows substantially into a long, folded tube before expanding into the short, curved, wide deferent duct. The deferent duct is ~ 2× as narrow as the prostate. The penis is unarmed. The vagina is long and narrow, slightly narrower than the deferent duct, and connects directly to the oval bursa copulatrix. The elongate seminal receptacle also connects to the bursa copulatrix next to the vaginal connection, and the short uterine duct, which enters the female gland complex. The bursa copulatrix is several times as large as the seminal receptacle (Fig. 3E).

Radular formula 13 × 19.0.19 in a 11-mm long specimen (MNHN IM-2013-86192). Rachidian teeth absent. Inner and mid-lateral teeth hamate, having a small cusp and lacking denticles (Fig. 4G–I). Innermost teeth very small in comparison to mid-laterals (Fig. 4G), elongate, with an inconspicuous secondary cusp mid-length. The teeth increase in size suddenly towards the medial portion of the half-row (Fig. 4G). Outermost teeth small, decreasing in size gradually, and hamate (Fig. 4I). No jaw was observed, labial cuticle smooth.

##### Biology.

Possibly a New Caledonia endemic, rare, 4–6 m depth. The single specimen was collected by dredging on coral debris and coarse sand bottoms.

##### Remarks.

*Phlegmodorispapillosa* Risbec, 1928 was originally described based on a single specimen collected in Nouméa, New Caledonia, with a short description and an illustration of the live animal. Risbec (1928) described the species as having the notum covered with large papillae and bearing spots with the appearance of black ocelli standing out against a yellowish background. Risbec (1928) also mentioned that the elongated, perfoliate rhinophores of *P.papillosa* are retractile in funnel-shaped sheaths with a well-marked ocelliform spotted papilla; and the gill is retractile in a cavity with a star-shaped orifice. The specimens here examined closely resemble the original description of *P.papillosa* with the exception that the notum is grey, not yellowish.

*Atagemaalbata* (Burn, 1962a) is a similar species, originally described as *Trippaalbata*, based on three specimens collected in Victoria, Australia. The specimens were described as pure white, sometimes with cream pigment, and characterized by having a soft, broad, flat body, with the mantle covered with low caryophyllidia, all similar in size, and with a mid-dorsal crest, extending from between the rhinophores to the branchial cavity. Burn (1962a) also described the branchial cavity as having an irregular outline and the rhinophores as perfoliate, with small, raised sheaths. Burn (1962a) compared *T.albata* with the New South Wales species *T.intecta* Kelaart, 1859 (= *Goniodoriserinaceus* Angas, 1864), which according to Burn (1962a) is usually much larger than *A.albata* and is of an ashy-brown color. With the available information is it not possible to confirm if *A.albata* and *A.papillosa* are the same species, and sequence data from *A.albata* would be needed to confirm this potential synonymy.

Finally, the specimen from the Philippines illustrated by Gosliner et al. (2018) as *Atagema* sp. 13 presents a similar external appearance and could be the same species. Examination of specimens is needed to confirm this possibility.

#### ﻿ Atagema kimberlyae sp. nov.

Taxon classificationAnimaliaNudibranchiaDiscodorididae

CE07FD12-0E0D-5B30-8653-BADC11B3B1F6

https://zoobank.org/FFE42C3A-F0E3-486D-9376-DC999DE7F241

Figs 2C, D , 3B, C , 4D–F

Atagema sp. 2: Hervé 2010: 190.

##### Type material.

***Holotype***: Koumac, New Caledonia (20°35.5'S, 164°16.4'E), 5 m depth [Koumac 2.1 stn. KR223, patch of sponges, small bits of sedimented coral, coarse sand and mud with algae], 19 Sep 2018, 20 mm long, dissected (MNHN IM-2013-86191, isolate JI43).

##### Description.

Body oval, flattened, covered with small, irregular tubercles and short ridges decreasing in size towards the mantle margin (Fig. 2C, D). A central, longitudinal area devoid of tubercles or ridges runs between the rhinophores and gill. A series of depressions on each side of the central ridge, generally decreasing in size towards the mantle margin. Entire dorsal surface, except for the depressions, covered with caryophyllidia. Branchial sheath composed of three large lobes; gill composed of five tripinnate branchial leaves, arranged horizontally in the living animal. Rhinophoral sheaths elevated; rhinophores long, lamellated, with 20 lamellae. Body color opaque greyish brown, with pale brown pigment mainly on top of the tubercles and ridges and scattered opaque white pigment; depressions dark brown to black (Fig. 2C). Rhinophores and branchial leaves are the same color as the dorsum.

Reproductive system (Fig. 3B, C) with a short, wide, simple ampulla that connects with the female gland complex and a convoluted prostate. The prostate has several folds and is approximately as wide as the ampulla. The prostate narrows slightly into a curved duct before expanding into the long, ovoid, wide deferent duct. At its widest point, the deferent duct is slightly wider than the prostate. The penis is unarmed. The vagina is long and narrow and connects directly to the spherical bursa copulatrix. The vagina is approximately as wide as the deferent duct. The small elongate seminal receptacle also connects to the bursa copulatrix near the vaginal connection and the short uterine duct that enters the female gland complex. The bursa copulatrix is several times larger than the seminal receptacle (Fig. 3C).

Radular formula 15 × 20.0.20 in a 20-mm long specimen (MNHN IM-2013-86191). Rachidian teeth absent. Inner and mid-lateral teeth hamate, having a small cusp and lacking denticles (Fig. 4D–F). Innermost teeth very small in comparison to mid-laterals (Fig. 4D), elongate, with an inconspicuous secondary cusp mid-length. The teeth increase in size suddenly towards the medial portion of the half-row (Fig. 4E). Outermost teeth small, decreasing in size gradually, and hamate (Fig. 4F). No jaw was observed, labial cuticle smooth.

##### Biology.

Possibly a New Caledonia endemic, rare, 5 m depth. The single specimen was obtained while SCUBA diving by direct collection on an unidentified sponge on which it was highly cryptic.

##### Etymology.

This species is named after Kimberly García Mendez, who participated in two of the Koumac expeditions, collecting a number of specimens and helping enormously with the processing and photographing of samples.

##### Remarks.

*Atagemakimberlyae* sp. nov. is assigned to the genus *Atagema* for two reasons, 1) the molecular phylogenetic analysis places the specimens sequenced in a clade containing *A.spongiosa*, a well stablished member of this genus (see above); 2) the morphological characteristics of this new species are consistent with the diagnosis of the genus provided by Valdés and Gosliner (2001), including a flexible body with series of tubercles all covered with caryophyllidia and depressions, the anterior border of the branchial sheath composed of three lobes and the gill leaves arranged horizontally; furthermore the prostate is tubular, with a single portion, the penis and vagina are unarmed, the labial cuticle smooth, and all radular teeth are hamate and smooth.

*Atagemakimberlyae* sp. nov. is morphologically similar to *Atagemaspongiosa* (described above), particularly to the juvenile specimens, but is genetically distinct. Also, it lacks the distinctive dorsal ridge of *A.spongiosa* and presents a number of anatomical differences, including a comparatively much shorter and wider ampulla, a wider prostate, a rounded bursa copulatrix instead of oval, and comparatively larger innermost lateral teeth. A review of the literature reveals that no other described Indo-Pacific species of *Atagema* are morphologically similar to *A.kimberlyae* sp. nov., hence it is described as new.

The geographic range of *Atagemakimberlyae* sp. nov. is close to that of *Atagemamolesta* (Miller, 1989 as *Trippamolesta*), introduced based on a single specimen collected from Te Hāwere-a-Maki (Goat Island), New Zealand. Miller (1989) described and illustrated the holotype, which differs from *A.kimberlyae* sp. nov. in several regards, including the more complex dorsal pattern of tubercles and ridges present in *A.molesta*, giving the animal a spikier appearance, and the reproductive system, which has a much larger deferent duct and a shorter prostate in *A.kimberlyae* sp. nov. While the radular morphology of the two species is similar, the radular formula is not, 23 × 32.0.32 in a 12-mm specimen of *A.molesta* versus 15 × 20.0.20 in a 20-mm long specimen of *A.kimberlyae* sp. nov.

Based on the species delimitation analysis presented here, *A.kimberlyae* sp. nov. is closely related but genetically distinct from specimens identified as Atagemacf.osseosa and *Atagemanotacristata* whose sequences are deposited in GenBank.

#### ﻿ Atagema sobanovae sp. nov.

Taxon classificationAnimaliaNudibranchiaDiscodorididae

DEDAF39A-D392-5E15-B90C-59876625D8C2

https://zoobank.org/C296974E-92C6-4F7C-8E4A-ABF61D48F896

Figs 5 , 6 , 7

?Atagema sp. 9: Gosliner et al. 2018: 116.

##### Type material.

***Holotype***: Koumac, New Caledonia (20°35.6'S, 164°16.3'E), 3 m depth [Koumac 2.1 stn. KR230], 28 Sep 2018, 22 mm long (MNHN IM-2013-86211, isolate JI16).

##### Other material examined.

Cap Deverd, Koumac, New Caledonia (20°46.2'S, 164°22.6'E), 5 m [Koumac 2.1 stn. KR213], 12 Sep 2018, 1 specimen 13 mm long (MNHN IM-2013-86178, isolate JI06). Koumac, New Caledonia (20°39.6'S, 164°16.2'E), 6 m depth [Koumac 2.1 stn. KR229], 27 Sep 2018, 1 specimen 8 mm long (MNHN IM-2013-86175, isolate JI28). Koumac, New Caledonia (20°35.6'S, 164°16.3'E), 3 m depth [Koumac 2.1 stn. KR230], 28 Sep 2018, 1 specimen 17 mm long, dissected (MNHN IM-2013-86180, isolate JI23); 1 specimen 18 mm long, dissected (MNHN IM-2013-86179, isolate JI21). Koumac, New Caledonia (20°35.1'S, 164°16.3'E), 7 m depth [Koumac 2.1 stn. KR409, muddy bottom with solitary soft and hard corals and hydroids], 28 Sep 2018, 1 specimen 8 mm long (MNHN IM-2013-86229). Koumac, New Caledonia (20°35.1'S, 164°16.3'E), 3 m [Koumac 2.2 stn. KR231], 1 Mar 2019, 1 specimen 9 mm long (MNHN IM-2013-86176, isolate JI44). Koumac, New Caledonia (20°37.3'S, 164°18'E), 6 m depth [Koumac 2.3 stn. KD522, grey sand with *Caulerpa* and *Halimeda*], 2 Nov 2019, 1 specimen 10 mm long (MNHN IM-2013-86174, isolate JI19). Koumac, New Caledonia (20°34.3'S, 164°13.5'E), 1–10 m depth [Koumac 2.3 stn. KR907, sanded slab with gorgonians, scattered seagrass, and *Caulerpa*; channel drop-off with gorgonians], 7 Nov 2019, 1 specimen 7 mm long (MNHN IM-2013-86173, isolate JI41). Koumac, New Caledonia (20°34.4'S, 164°13.8'E), 8 m depth [Koumac 2.3 stn. KR913], 14 Nov 2019, 1 specimen 11 mm long (MNHN IM-2013-86171, isolate JI29); 1 specimen 10 mm long (MNHN IM-2013-86172, isolate JI46). Koumac, New Caledonia (20°35.1'S, 164°16.3'E), 7 m depth [Koumac 2.3 stn. KR1019, “fond de vase” with *Caulerpa* and sponges], 4 Nov 2019, 1 specimen 28 mm long (MNHN IM-2013-86181, isolate JI05); 1 specimen 22 mm long, dissected (MNHN IM-2013-86187, isolate JI08); 1 specimen 9 mm long (MNHN IM-2013-86177, isolate JI45).

##### Description.

Body oval, elevated, completely covered with a dense, complex network of delicate ridges (Fig. 5). Large caryophyllidia present at the points where ridges meet. A series of small depressions free of ridges and caryophyllidia present on each side of the mantle. A single, elevate dorsal hump present on the center of the dorsum, not visible in juvenile specimens (Fig. 5E). Branchial sheath composed of three lobes; gill composed of five tripinnate branchial leaves, arranged horizontally in the living animal. Rhinophoral sheaths elevated; rhinophores long, lamellated, with 8–10 lamellae. Body color opaque creamy grey, depressions a bit darker. Rhinophores and branchial leaves are the same color as the dorsum.

**Figure 6.**
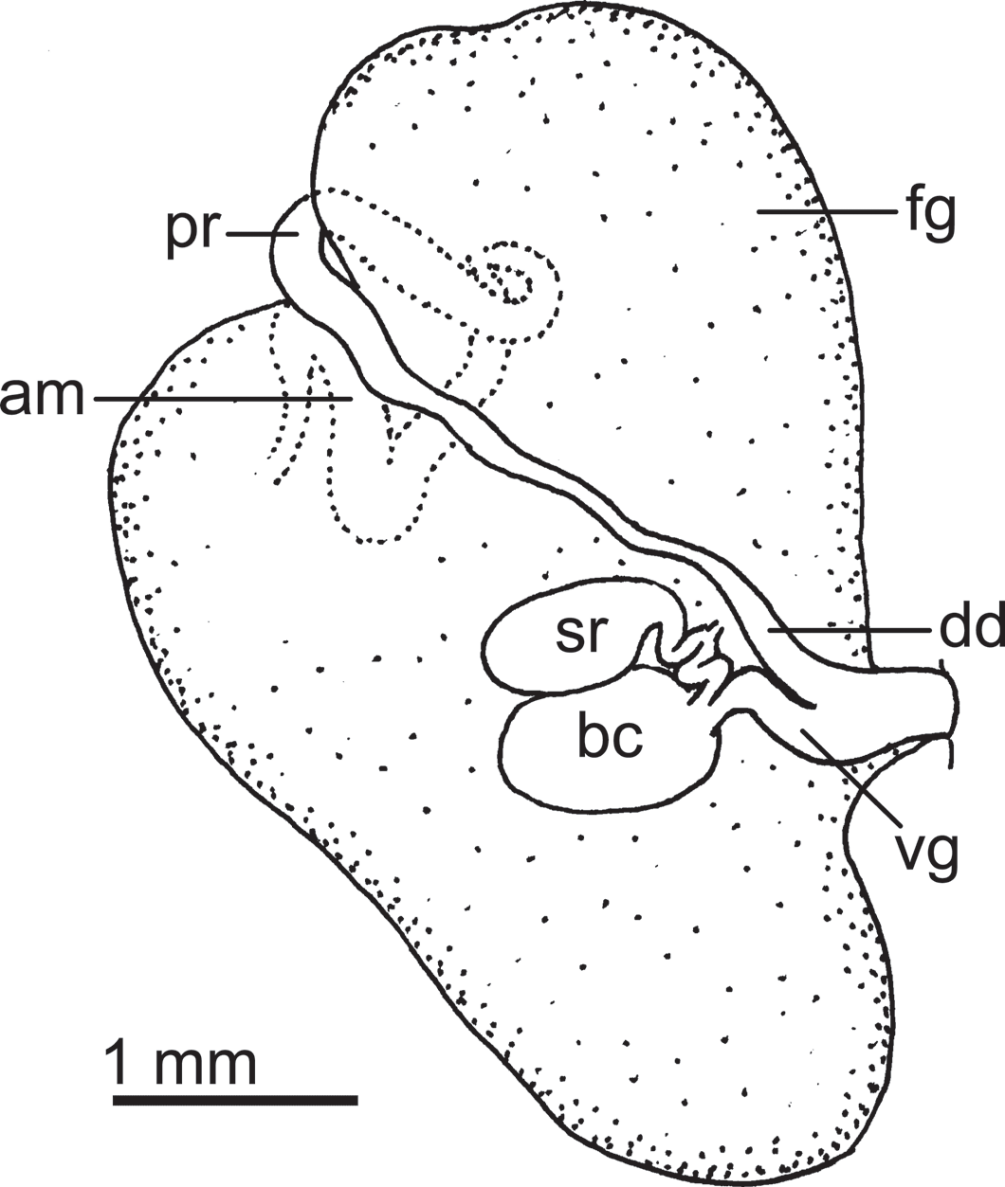
Drawing of the reproductive system of *Atagemasobanovae* sp. nov., MNHN IM-2013-86180. Abbreviations: am, ampulla; bc, bursa copulatrix; dd, deferent duct; fg, female gland complex; pr, prostate; sr, seminal receptacle; vg, vagina.

Reproductive system (Fig. 6) with a short, convoluted ampulla that connects with the female gland complex and an elongated prostate. The prostate is as long as the ampulla and it narrows slightly into an elongate duct before expanding into the short, simple, deferent duct. The penis is unarmed. The vagina is short and wide, approximately as wide as the deferent duct, and connects directly to the oval bursa copulatrix. The oval seminal receptacle also connects to the bursa copulatrix next to the vaginal opening and the short uterine duct that enters the female gland complex. The bursa copulatrix is slightly larger than the seminal receptable.

**Figure 7.**
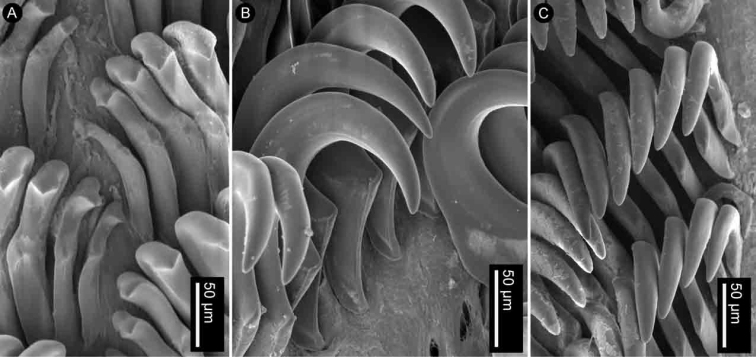
SEM of the radula of specimens of *Atagemasobanovae* sp. nov., MNHN IM-2013-86179, innermost teeth (**A**), mid-lateral teeth (**B**), outer lateral teeth (**C**).

Radular formula 22 × 35.0.35 in an 18-mm long specimen (MNHN IM-2013-86179) and 17 × 34.0.34 in a 22-mm long specimen (MNHN IM-2013-86187). Rachidian teeth absent. Inner and mid-lateral teeth hamate, having a small cusp and lacking denticles (Fig. 7A, B). Innermost teeth very small in comparison to mid-laterals (Fig. 7A). The teeth increase in size gradually towards the medial portion of the half-row (Fig. 7A). Outermost teeth small, decreasing in size gradually, and hamate (Fig. 7C). No jaw was observed, labial cuticle smooth.

##### Biology.

This species could be widespread in the Western Pacific (see remarks). Found in shallow water (1–10 m depth). The specimens were exclusively collected on an unidentified species of grey sponge inhabiting the surface of scallops; the nudibranchs were highly cryptic on the sponge and often found buried in the sponge tissue. Few specimens were obtained by direct collection while SCUBA diving but more of them were found in the lab while searching for crustaceans associated with the sponges.

##### Etymology.

This species is named after Anna Šobáňová, crustacean expert who originally discovered this species in the field while looking for crustaceans living in sponges.

##### Remarks.

*Atagemasobanovae* sp. nov. is assigned to the genus because of its position in the molecular phylogenetic trees, in a clade containing other species of *Atagema* such as *A.spongiosa* and A.cf.osseosa. Also, the morphological characteristics of this new species are consistent with the diagnosis of the genus by Valdés and Gosliner (2001). *Atagemasobanovae* sp. nov. has a flexible body with series of dorsal ridges and a central conspicuous tubercle, all covered with caryophyllidia, the anterior border of the branchial sheath is composed of three lobes and the gill is arranged horizontally; the prostate is tubular, with a single portion, the penis and vagina are unarmed; the labial cuticle smooth and all radular teeth are hamate and smooth.

A review of the literature shows that no other described species of *Atagema* possesses the external characteristics of *A.sobanovae* sp. nov. The only other tropical Indo-Pacific species with a uniform color is *Atagemacarinata* (Quoy & Gaimard, 1832), which was described from the coast of Thames, New Zealand, as yellowish white with a dorsal longitudinal ridge between the rhinophores and the gill. The illustration provided by Quoy and Gaimard (1832–1833: pl. 16, figs 10–14) represents an animal with a distinct dorsal ridge very different from the complex dorsal pattern of *A.sobanovae* sp. nov. with depressions, ridges, and a central tubercle. The specimens of *A.carinata* described and illustrated by Rudman (2005) are consistent with the original description.

A specimen from the Philippines illustrated by Gosliner et al. (2018) as *Atagema* sp. 9 could belong to *A.sobanovae* sp. nov. but this needs anatomical and molecular confirmation.

#### ﻿Genus Jorunna

Taxon classificationAnimaliaNudibranchiaDiscodorididae

Bergh, 1876

14ED5D55-0418-50F6-8A6C-0AF3659AC676

Kentrodoris Bergh, 1876: 413. Type species: Kentrodorisrubescens Bergh, 1876 [= Jorunnarubescens Bergh, 1876], by subsequent designation by Ev. Marcus (1976).
Jorunna Bergh, 1876: 414. Type species: Dorisjohnstoni Alder & Hancock, 1845 [= Jorunnatomentosa (Cuvier, 1804)], by monotypy.
Audura Bergh, 1878: 567–568. Type species: Auduramaima Bergh, 1878 [= Jorunnamaima (Bergh, 1878)], by monotypy.
Centrodoris P. Fischer 1880–1887 [1883]: 522 (unjustified emendation for Kentrodoris Bergh, 1876).
Awuka Er. Marcus, 1955: 155–156. Type species Awukaspazzola Er. Marcus, 1955 [= Jorunnaspazzola (Er. Marcus, 1955)], by original designation.

##### Remarks.

For an in-depth discussion of the characteristics of the genus *Jorunna* and its synonyms see Camacho-García and Gosliner (2008).

#### ﻿ Jorunna daoulasi sp. nov.

Taxon classificationAnimaliaNudibranchiaDiscodorididae

480244CE-1167-5354-8F6F-05876F07EC1C

https://zoobank.org/0D41E0FA-826A-4761-BB7B-B71CE0B2E97E

**Figure 8.**
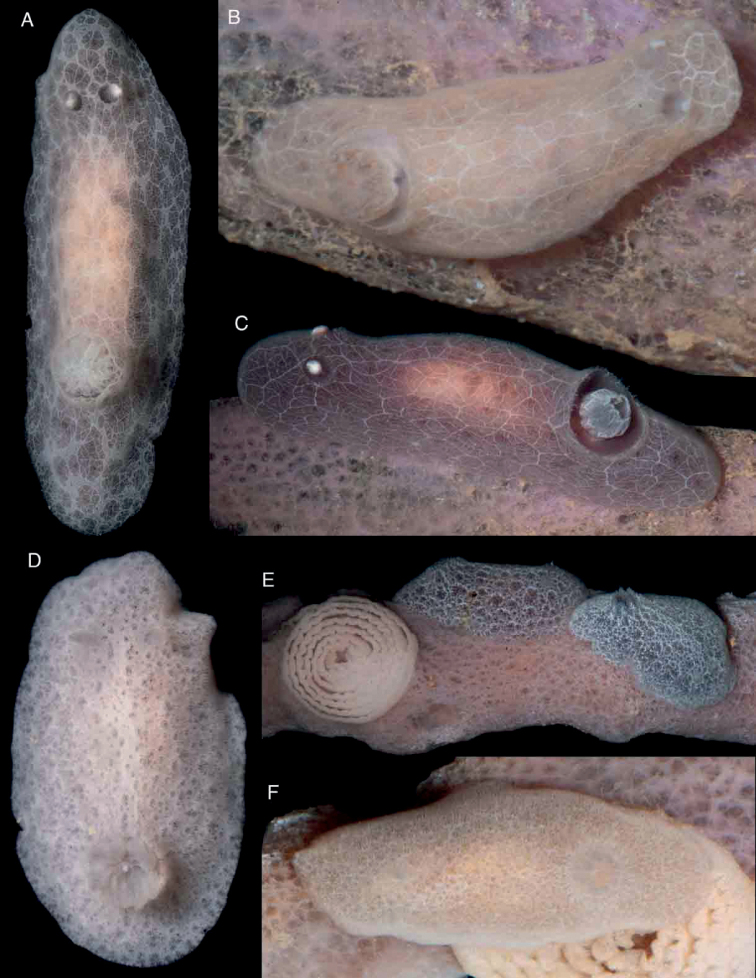
Photographs of live animals of the genus *Jorunna* Bergh, 1876 **A–C***Jorunnadaoulasi* sp. nov., MNHN IM-2013-86219 on black background (**A**), MNHN IM-2013-86220 in situ (**B**), Holotype (MNHN IM-2013-86230) in situ (**C**) **D–F***Jorunnahervei* sp. nov., MNHN IM-2013-86228 on black background (**D**), MNHN IM-2013-86224 and Holotype MNHN IM-2013-86225 in situ with egg mass (**E**), MNHN IM-2013-86226 in situ with egg mass (**F**).

**Figure 9.**
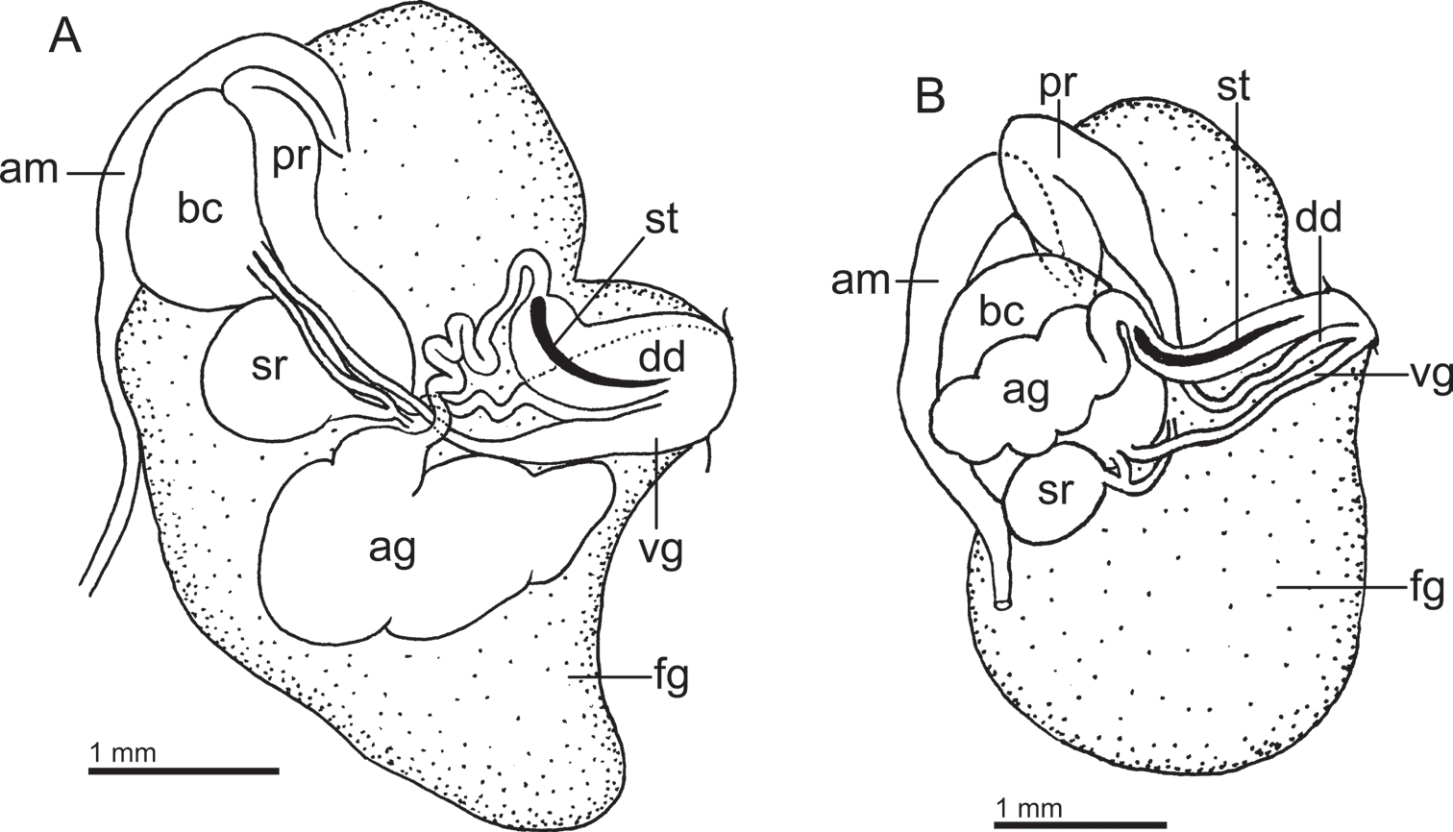
Drawings of the reproductive systems of specimens of the genus *Jorunna* Bergh, 1876 **A***Jorunnadaoulasi* sp. nov., MNHN IM-2013-86219 **B***Jorunnahervei* sp. nov., MNHN IM-2013-86226. Abbreviations: ag, accessory gland; am, ampulla; bc, bursa copulatrix; dd, deferent duct; fg, female gland complex; pr, prostate; sr, seminal receptacle; st, stylet; vg, vagina.

**Figure 10.**
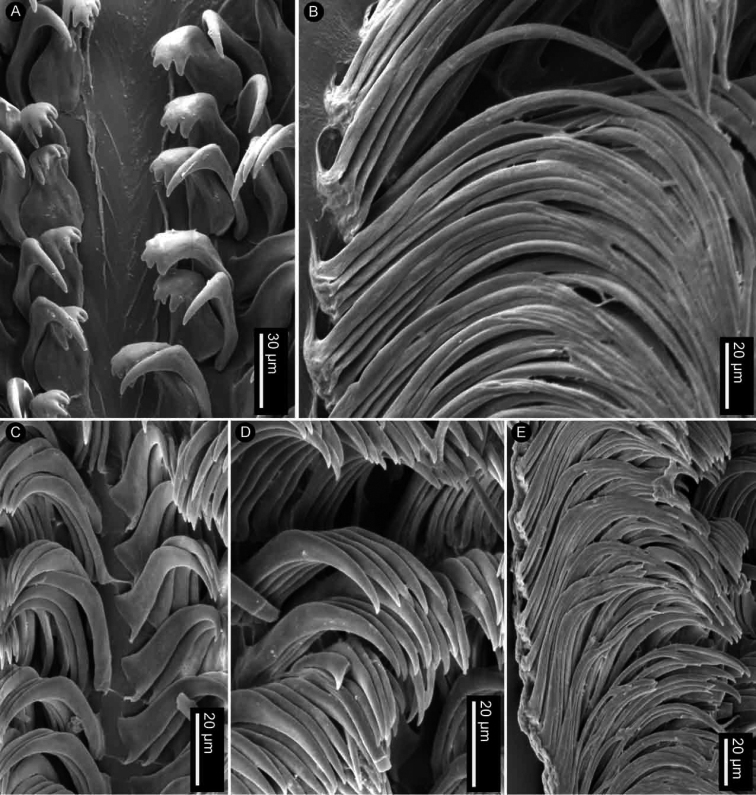
SEM of the radula of specimens of the genus *Jorunna* Bergh, 1876 **A, B***Jorunnadaoulasi* sp. nov., MNHN IM-2013-86220, innermost teeth (**A**), outer lateral teeth (**B**) **C–E***Jorunnahervei* sp. nov., MNHN IM-2013-86224, innermost teeth (**C**), mid-lateral teeth (**D**), outer lateral teeth (**E**).

Figs 8A–C , 9A , 10A, B

?Jorunna sp. 10: Gosliner et al. 2018: 122.
?Rostanga sp. 4: Nakano 2018: 263.

##### Type material.

***Holotype***: In front of the harbor, Koumac, New Caledonia (20°35.3'S, 164°16.4'E), 6 m depth [Koumac 2.1 stn. KR220], 17 Nov 2018, 12 mm long, (MNHN IM-2013-86230).

##### Other material examined.

In front of the harbor, Koumac, New Caledonia (20°35.3'S, 164°16.4'E), 6 m depth [Koumac 2.1 stn. KR220], 17 Nov 2018, 1 specimen 24 mm long, dissected (MNHN IM-2013-86220). Koumac, New Caledonia (20°35.2'S, 164°16.3'E), 6 m depth [Koumac 2.3 stn. KR886], 21 Nov 2019, 1 specimen 27 mm long, dissected (MNHN IM-2013-86219, isolate JI22).

##### Description.

Body oval, narrow, elongate, completely covered with numerous caryophyllidia (Fig. 8A–C). Branchial and rhinophoral sheaths low, simple, circular; gill composed of nine short, tripinnate branchial leaves, imbricated, arranged upright, with the apices close to each other in the living animal. Rhinophores short, lamellated, with eight or nine lamellae. Body color grey, with a complex network of white lines of different thicknesses; in some specimens some of the lines are very thick and contain darker areas (Fig. 8A), whereas in others thicker lines form the main network and thinner lines form a secondary network (Fig. 8) and in others all lines are approximately the same thickness (Fig. 8B). Rhinophores and branchial leaves are the same color as the dorsum but the rhinophoral lamellae and in some cases the gill lamellae are white.

Reproductive system (Fig. 9A) with a long, narrow, curved ampulla that connects with the female gland complex and an elongate prostate. The prostate is as wide as the ampulla but narrows substantially before expanding into the short, curved, narrow deferent duct. The deferent duct is much narrower than the prostate. The penis is unarmed. The vagina is very elongate and wide distally, several times wider than the deferent duct, narrowing considerably proximally and connecting directly to the irregular bursa copulatrix. The oval seminal receptacle also connects to the bursa copulatrix next to the vaginal connection, and the short uterine duct that enters the female gland complex. The bursa copulatrix is ~ 3× as large as the seminal receptable. A large accessory gland connects to a narrow and convoluted duct that opens into the genital atrium, where a curved, sharp stylet is located.

Radular formula 24 × n.0.n in a 26-mm long specimen (MNHN IM-2013-86220) and 25 × n.0.n in a 27-mm long specimen (MNHN IM-2013-86219). Rachidian teeth absent. Innermost lateral teeth wide, having a short cusp with four or five irregular denticles (Fig. 10A). Mid-lateral teeth hamate, lacking denticles (Fig. 10A). The teeth increase in size gradually towards the distal portion of the half-row (Fig. 10B). Outermost teeth very elongate, longer than mid-lateral teeth, with several elongate apical denticles (Fig. 10B). No jaws ware observed.

##### Biology.

Range includes New Caledonia and possibly Papua New Guinea and Japan (see Remarks section below); uncommon, found at ~ 6 m depth on an unidentified grey sponge on which it is highly cryptic. All the specimens were found directly on the sponges while SCUBA diving.

##### Etymology.

This species is named after Alain Daoulas, outstanding collector and naturalist, who participated in two of the Koumac expeditions, collecting a number of important specimens.

##### Remarks.

*Jorunnadaoulasi* sp. nov. is placed in the genus *Jorunna* because it fits morphologically within the diagnoses of the genus provided by Valdés and Gosliner (2001) and Camacho-García and Gosliner (2008). Specifically, *J.daoulasi* sp. nov. has a soft mantle covered with long caryophyllidia, the radular teeth are hamate, and the reproductive system has an accessory gland and a copulatory stylet. Finally, in the molecular phylogenetic analyses, *J.daoulasi* sp. nov. is a member of a well-supported clade containing other members of *Jorunna*.

Camacho-García and Gosliner (2008) provided a comprehensive revision and illustrations of the valid species of the genus *Jorunna*, including all the Indo-Pacific taxa described to date. None of the species included in Camacho-García and Gosliner’s (2008) monograph have a similar color pattern and morphology to *J.daoulasi* sp. nov. Since then, several additional new species have been described from the Atlantic Ocean (Edmunds 2011; Alvim and Pimenta 2013; Ortea et al. 2014; Ortea and Moro 2016; Neuhaus et al. 2021) and the Indian Ocean (Tibiriçá et al. 2023), but they are also morphologically and/or genetically different from *J.daoulasi* sp. nov. The most similar species to *J.daoulasi* sp. nov. in external morphology are *Jorunna* sp. 10 from Papua New Guinea illustrated by Gosliner et al. (2018) and *Rostanga* sp. 4 from Japan illustrated by Nakano (2018), which have a very similar body shape and color and could represent the same species.

#### ﻿ Jorunna hervei sp. nov.

Taxon classificationAnimaliaNudibranchiaDiscodorididae

E48443F4-0CD3-5DDB-ADCC-62EFC8EEA3A1

https://zoobank.org/DAD18B3C-3AFA-428E-909E-71309CE1ACB3

Figs 8D–F , 9B , 10C–E

##### Type material.

***Holotype***: Pandop, Koumac, New Caledonia (20°34.9'S, 164°16.5'E), 7 m depth [Koumac 2.1 stn. KR868, rock, sponges, algae including *Halimeda*], 26 Sep 2018, 1 specimen 24 mm long (MNHN IM-2013-86225, isolate JI47)

##### Other material examined.

Koumac, New Caledonia (20°35.6'S, 164°16.3'E), 3 m depth [Koumac 2.1 stn. KR230], 28 Sep 2018, 1 specimen 11 mm long (MNHN IM-2013-86221). Koumac, New Caledonia (20°35.1'S, 164°16.3'E), 3 m depth [Koumac 2.1 stn. KR231], 29 Sep 2018, 1 specimen 21 mm long, dissected (MNHN IM-2013-86222). Koumac, New Caledonia (20°35.1'S, 164°16.2'E), 8 m depth [Koumac 2.1 stn. KR410, sponge bottom], 29 Sep 2018, 1 specimen 14 mm long (MNHN IM-2013-86223). Pandop Point Reef, Koumac, New Caledonia (20°35.2'S, 164°16.3'E), 6 m depth [Koumac 2.1 stn. KR859, sandy-muddy bottom with sponges, *Caulerpa*], 17 Sep 2018, 1 specimen 25 mm long, dissected (MNHN IM-2013-86226, isolate JI48); 1 specimen 14 mm long (MNHN IM-2013-86227). Pointe de Pandop, Koumac, New Caledonia (20°34.9'S, 164°16.5'E), 7 m depth [Koumac 2.1 stn. KR868, rock, sponges, algae including *Halimeda*], 26 Sep 2018, 1 specimen 22 mm long (MNHN IM-2013-86224). Koumac, New Caledonia (20°32.9'S, 164°16.8'E), 5 m depth [Koumac 2.3 stn. KR917], 19 Nov 2019, 1 specimen 16 mm long (MNHN IM-2013-86228).

##### Description.

Body oval, flattened, completely covered with numerous caryophyllidia (Fig. 8D–F). Branchial and rhinophoral sheaths low, simple, circular; gill composed of nine short, tripinnate branchial leaves, slightly imbricated, arranged fully upright in the living animal. Rhinophores short, lamellated with elongate apices, seven or eight lamellae. Body color variable from pale brown to grey, with numerous irregular dark patches, surrounded by white pigment (Fig. 8E). Rhinophores and branchial leaves are the same color as the dorsum.

Reproductive system (Fig. 9B) with an elongate, curved ampulla that connects with the female gland complex and an elongate prostate with a single fold. The prostate is as wide as the ampulla but narrows substantially into a long tube before expanding slightly into the short, curved, narrow deferent duct. The penis is unarmed. The vagina is narrow, as wide as the deferent duct, and very elongate, connecting directly to the oval bursa copulatrix. The oval seminal receptacle also connects to the bursa copulatrix next to the vaginal connection, and the long uterine duct that enters the female gland complex. The bursa copulatrix is many times larger than the seminal receptacle. A large accessory gland connects to a wide duct that opens into the genital atrium, where a sharp, curved stylet is located.

Radular formula 24 × n.0.n, in a 21-mm long specimen (MNHN IM-2013-86222), 28 × n.0.n in a 22-mm long specimen (MNHN IM-2013-86224), and 30 × n.0.n in a 25-mm long specimen (MNHN IM-2013-86226). Rachidian teeth absent. Inner and mid-lateral teeth hamate, having a long cusp and lacking denticles (Fig. 10C–E). Innermost teeth smaller than mid-laterals (Fig. 10C). The teeth increase in size gradually towards the medial portion of the half-row (Fig. 10D). Outermost teeth very elongate, longer than mid-lateral teeth, increasing in size gradually, and hamate (Fig. 10E). No jaws were observed.

##### Biology.

The pale brown egg mass is a highly coiled ribbon with ca. seven tightly packed whorls with a wavy upper edge (Fig. 8E). Eggs are ~ 105 µm in diameter. The geographic range includes New Caledonia and could be an endemic species; uncommon, found at 3–8 m depth on an unidentified brownish grey sponge on which is highly cryptic. All the specimens were collected directly from the sponges while SCUBA diving.

##### Etymology.

This species is named after Jean-François Hervé, pioneer in the study of the sea slugs of New Caledonia and excellent collector; he participated in two of the Koumac expeditions, finding numerous specimens.

##### Remarks.

As in the case of *Jorunnadaoulasi* sp. nov., *Jorunnahervei* sp. nov. is placed in the genus *Jorunna* because it fits morphologically within the diagnoses of the genus provided by Valdés and Gosliner (2001) and Camacho-García and Gosliner (2008). *Jorunnahervei* sp. nov. has a soft mantle covered with long caryophyllidia, the radular teeth are hamate, and the reproductive system has an accessory gland and a copulatory stylet, all of which are characteristics of *Jorunna*. Furthermore, in the molecular phylogenetic analyses, *Jorunnahervei* sp. nov. is sister to *J.daoulasi* sp. nov. as well as a member of a well-supported clade containing other members of *Jorunna*.

*Jorunnahervei* sp. nov. differs from *Jorunnadaoulasi* sp. nov. in several regards. Externally, *J.hervei* sp. nov. is less elongate than *J.daoulasi* sp. nov. and lacks the network of white pigment; instead it has numerous irregular dark patches, in some specimens surrounded by white pigment. The reproductive system of *J.hervei* sp. nov. is similar to that of *J.daoulasi* sp. nov., but the accessory gland is comparatively smaller, the bursa copulatrix is much larger in comparison to the seminal receptable, and the deferent duct is shorter in comparison to the vagina. The main anatomical difference between these two species is the radular morphology, while *Jorunnahervei* sp. nov. has inner and mid-lateral teeth hamate, having a long cusp and lacking denticles, in *J.daoulasi* sp. nov. the innermost lateral teeth are wide, having a short cusp with four or five irregular denticles. Finally, the ABGD analysis recovered *J.hervei* sp. nov. and *J.daoulasi* sp. nov. as distinct species.

*Jorunnaliviae* Tibiriçá, Strömvoll & Cervera, 2023 recently described from Mozambique (Tibiriçá et al. 2023) is sister to *J.hervei* sp. nov. and is morphologically similar but differs in several important respects. First of all, the species delimitation analysis recovered *J.hervei* sp. nov. and *Jorunnaliviae* as different species. Additionally, the body of *J.liviae* appears to be narrower and more elongate than that of *J.hervei* sp. nov. More importantly, the outermost radular teeth of *J.liviae* contain multiple elongate denticles, which are absent in all specimens examined of *J.hervei* sp. nov. Also, the prostate of *J.liviae* is flattened, whereas the prostate of *J.hervei* sp. nov. is tubular an elongate, and the accessory gland appears to be comparatively much larger in *J.liviae* than in *J.hervei* sp. nov. although is it variable in size (Tibiriçá et al. 2023). Finally, the eggs of *J.liviae* are white, whereas they are pale brown in *J.hervei* sp. nov. It is clear that these two species are similar but distinct.

A review of the literature does not reveal any other species morphologically similar to *J.hervei* sp. nov. *Rostanga* sp. 7 in Gosliner et al. (2018) has some superficial resemblance but there are some obvious differences, including the background color, grey in *J.hervei*, pink in *Rostanga* sp. 7, and the egg mass, having one or two loosely packed whorls with ochre, large eggs in *Rostanga* sp. 7, versus seven tightly packed whorls with pale brown eggs in *J.hervei*.

#### ﻿Genus Rostanga

Taxon classificationAnimaliaNudibranchiaDiscodorididae

Bergh, 1879

3BCFA617-C388-519D-B428-0947E59EDF7E

Rostanga Bergh, 1879: 353–354. Type species: Doriscoccinea Forbes in Alder & Hancock, 1848 [= Rostangarubra (Risso, 1818)], by original designation.
Boreodoris Odhner, 1939: 31–33. Type species: Boreodorissetidens Odhner, 1939 [= Rostangasetidens (Odhner, 1939)], by monotypy.
Rhabdochila P. Fischer, 1880–1887 [1883]: 521. Type species Doriscoccinea Forbes in Alder & Hancock, 1848 [= Rostangarubra (Risso, 1818)], by subsequent designation by Iredale and O’Donoghue (1923).

##### Remarks.

For an in-depth discussion of the characteristics of the genus *Rostanga* and its synonyms see Rudman and Avern (1989) and Valdés and Gosliner (2001).

#### ﻿ Rostanga poddubetskaiae sp. nov.

Taxon classificationAnimaliaNudibranchiaDiscodorididae

F3330D3A-2D81-59F8-9D11-D6BBA56FFC21

https://zoobank.org/EF949405-58D8-4D48-AD09-1CB3CE3993F5

Figs 11 , 12 , 13

##### Type material.

***Holotype***: Anse de Koumac, Koumac, New Caledonia (20°34'S, 164°16'E), 4 m depth [Koumac 2.1 stn. KR206], 5 Sep 2018, 1 specimen 23 mm long (MNHN IM-2013-86199, isolate JI01).

##### Other material examined.

Anse de Koumac, Koumac, New Caledonia (20°34'S, 164°16'E), 4 m depth [Koumac 2.1 stn. KR206], 5 Sep 2018, 1 specimen 25 mm long (MNHN IM-2013-86200, isolate JI17); 1 specimen 12 mm long (MNHN IM-2013-86201, isolate JI32); 1 specimen 26 mm long, dissected (MNHN IM-2013-86202, isolate JI03); 1 specimen 19 mm long, dissected (MNHN IM-2013-86203, isolate JI12); 1 specimen 16 mm long, dissected (MNHN IM-2013-86204, isolate JI20). Cap Deverd, Koumac, New Caledonia (20°46.2'S, 164°22.6'E), 5 m depth [Koumac 2.1 stn. KR213], 29 Sep 2018, 1 specimen 26 mm long, dissected (MNHN IM-2013-86205, isolate JI27); 1 specimen 28 mm long, dissected (MNHN IM-2013-86206, isolate JI13). Anse de Koumac, Koumac, New Caledonia (20°34.6'S, 164°16.1'E), 5 m depth [Koumac 2.1 stn. KR219], 17 Sep 2018, 1 specimen 12 mm long (MNHN IM-2013-86207, isolate JI39); 1 specimen 23 mm long, dissected (MNHN IM-2013-86208, isolate JI25); 1 specimen 26 mm long (MNHN IM-2013-86209, isolate JI18); 1 specimen 17 mm long (MNHN IM-2013-86210, isolate JI40). Koumac, New Caledonia (20°35.6'S, 164°16.3'E), 3 m depth [Koumac 2.2 stn. KR230], 2 Mar 2019, 1 specimen 20 mm long (MNHN IM-2013-86213, isolate JI36); 2 Mar 2019, 1 specimen 21 mm long (MNHN IM-2013-86214, isolate JI37); 3 Mar 2019, 1 specimen 20 mm long (MNHN IM-2013-86212, isolate JI31). Pointe de Pandop, Koumac, New Caledonia (20°34.9'S, 164°16.5'E), 7 m depth [Koumac 2.1 stn. KR868], 26 Sep 2018, 1 specimen 26 mm long (MNHN IM-2013-86215, isolate JI15); 1 specimen 24 mm long (MNHN IM-2013-86216, isolate JI24); 1 specimen 14 mm long (MNHN IM-2013-86217, isolate JI38). Koumac, New Caledonia (20°33.7'S, 164°13.1'E), 12 m depth [Koumac 2.3 stn. KR206], 3 Nov 2019, 1 specimen 19 mm long (MNHN IM-2013-86218, isolate JI07).

##### Description.

**Figure 11.**
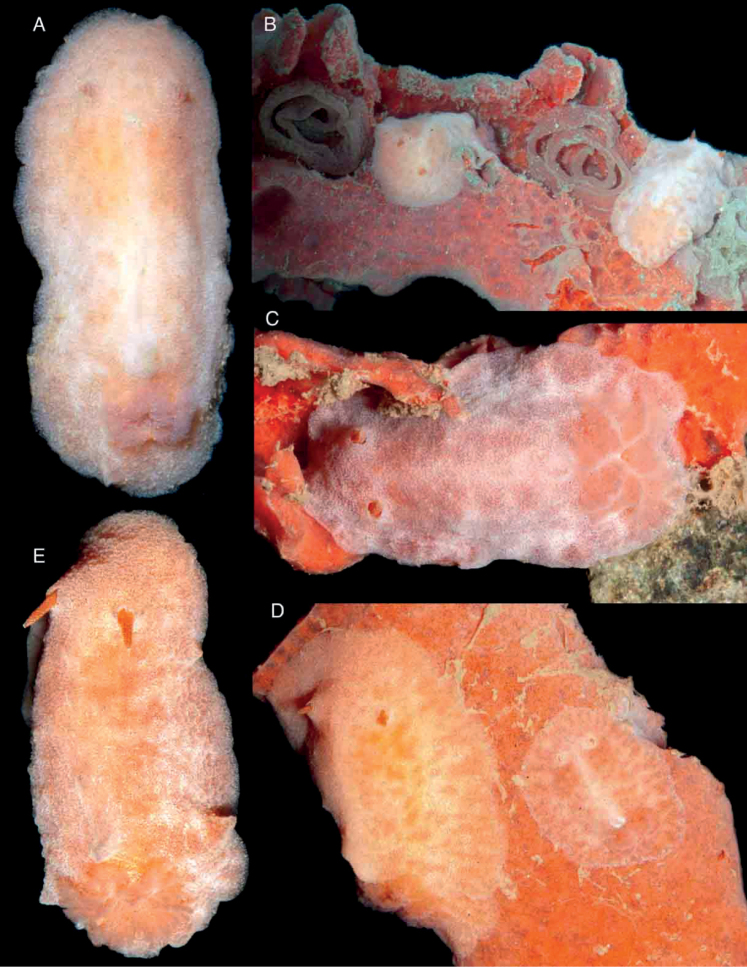
Photographs of live animals of *Rostangapoddubetskaiae* sp. nov. **A** holotype MNHN IM-2013-86199 on black background **B** holotype (MNHN IM-2013-86199) and MNHN IM-2013-86217 in situ with egg masses **C**MNHN IM-2013-86205 in situ **D**MNHN IM-2013-86216 and MNHN IM-2013-86217 in situ **E**MNHN IM-2013-86209 on black background.

Body oval, elongate, completely covered with numerous caryophyllidia (Fig. 11). Branchial and rhinophoral sheaths low, simple, circular; gill composed of seven wide, tripinnate branchial leaves, extended laterally, lying on the dorsum in the living animal. A low, irregular, inconspicuous ridge runs between the rhinophores and the gill, not clearly visible in all specimens. Rhinophores very elongate, almost conical, lamellated, with 15 or 16 lamellae. Body color pinkish to orange, with irregular darker patches all over the dorsum. Rhinophores reddish; branchial leaves the same color as the dorsum.

**Figure 12.**
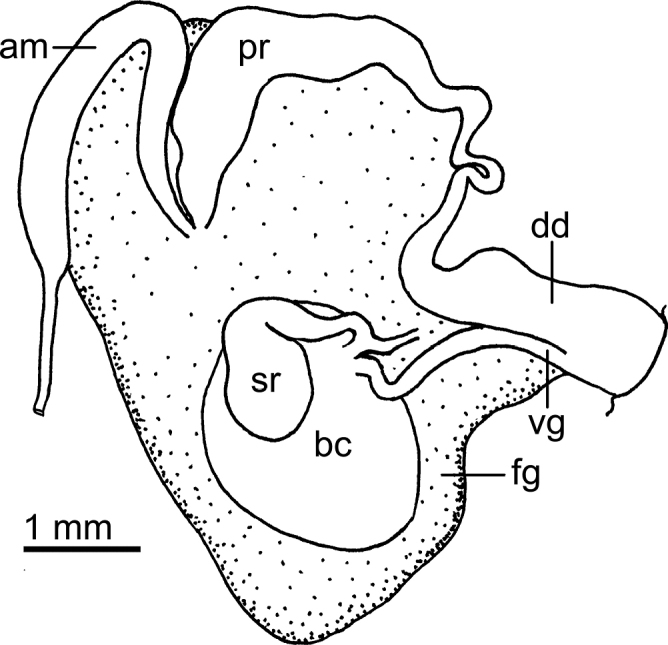
Drawing of the reproductive system of *Rostangapoddubetskaiae* sp. nov., MNHN IM-2013-86202. Abbreviations: am, ampulla; bc, bursa copulatrix; dd, deferent duct; fg, female gland complex; pr, prostate; sr, seminal receptacle; vg, vagina.

Reproductive system (Fig. 12) with a long, narrow, curved ampulla that connects with the female gland complex and an irregular, elongate prostate. The prostate is wider than the ampulla, but it narrows substantially into a long, folded tube, before expanding into the short, wide deferent duct. The penis is unarmed. The vagina is elongate, several times narrower than the deferent duct, connecting directly to the large, oval bursa copulatrix. The smaller, elongate seminal receptacle also connects to the bursa copulatrix next to the vaginal connection, and the short uterine duct that enters the female gland complex. The bursa copulatrix is several times larger than the seminal receptacle.

**Figure 13.**
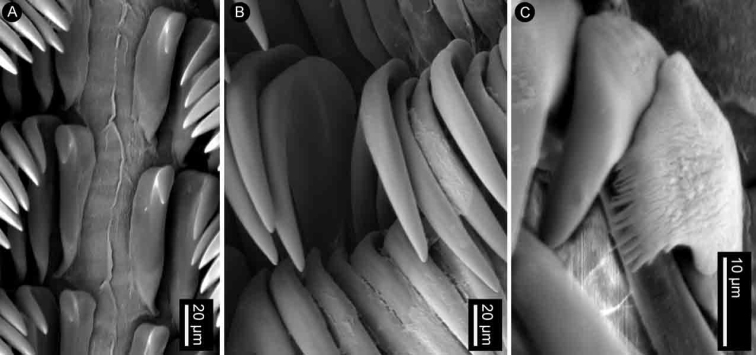
SEM of the radula of specimens of *Rostangapoddubetskaiae* sp. nov., MNHN IM-2013-86205 **A** innermost teeth **B** mid-lateral teeth **C** outer lateral teeth.

Radular formula 28 × 73.0.73 in a 23-mm long specimen (MNHN IM-2013-86208), 36 × 80.0.80 in a 26-mm long specimen (MNHN IM-2013-86205), and 37 × 81.0.81 in a 26-mm long (MNHN IM-2013-86209). Rachidian teeth absent. Inner and mid-lateral teeth hamate, having a small cusp and lacking denticles (Fig. 13A, B). Innermost teeth very small in comparison to mid-laterals (Fig. 13A). The teeth increase in size gradually towards the medial portion of the half-row (Fig. 13B). Outermost teeth small, decreasing in size gradually, and hamate (Fig. 13C), outermost one with 13–20 irregular denticles. No jaw was observed, labial cuticle smooth.

##### Biology.

All the specimens were found on an unidentified species of sponge while SCUBA diving. The presence of these highly cryptic nudibranchs was initially determined in the field by observing the egg masses on the sponges. In most cases, to separate the nudibranchs, the sponges were brought to the lab and examined under a microscope.

##### Etymology.

This species is named after Marina Poddubetskaia, indefatigable collector and diver, who first discovered the animals here described during the two of the Koumac expeditions.

##### Remarks.

*Rostangapoddubetskaiae* sp. nov. is provisionally assigned to the genus *Rostanga* based on the results of the molecular phylogenetic analyses, which place this species solidly nested within a clade containing other species identified as members of *Rostanga*. However, there are some notable differences between *Rostangapoddubetskaiae* sp. nov. and the diagnoses of the genus *Rostanga* provided by Rudman and Avern (1989) and Valdés and Gosliner (2001), such as the absence of jaws and elongate outermost radular teeth, and the presence of short caryophyllidia; moreover, the arrangement of the branchial leaves flattened against the dorsum and the presence of a dorsal ridge are unusual for a species of *Rostanga*. Additional resolution in the phylogeny of dorid nudibranchs and a larger sample are needed before this species can be placed in a genus with confidence.

*Rostangapoddubetskaiae* sp. nov. appears to be sister to *Rostangaelandsia* Garovoy, Valdés & Gosliner, 2001 from South Africa, but additional species need to be included in the analysis to confirm those relationships. Morphologically, *R.poddubetskaiae* sp. nov. exhibits a number of differences from other members of this genus, including the presence of a dorsal ridge, elongate rhinophores, a gill flattened against the body, and smooth, hamate inner and mid radular teeth, and short, pectinate outermost lateral teeth. The Indo-Pacific species of *Rostanga* have been reviewed in papers by Rudman and Avern (1989), Baba (1991), and Garovoy et al. (2001), and none of them have external and internal characteristics present in *R.poddubetskaiae* sp. nov. The only exception is *Rostangacrawfordi* (Burn, 1969), described as *Rostangaaustralis* Rudman & Avern, 1989, which appears to have a dorsal ridge in some specimens (see Rudman and Avern 1989; Coleman 2008) and a similar external coloration to *R.poddubetskaiae* sp. nov., but the radular teeth are very different: specifically, the outer teeth are elongate with numerous denticles on the tip.

#### ﻿Genus Sclerodoris

Taxon classificationAnimaliaNudibranchiaDiscodorididae

Eliot, 1904

F9D89CF8-A249-5DF9-AC41-286790663BD6

Sclerodoris Eliot, 1904: 361. Type species: Sclerodoristuberculata Eliot, 1904, by subsequent designation by Valdés and Gosliner (2001).
?Gravieria Vayssière, 1912: 29–30. Type species: Gravieriarugosa Vayssière, 1912, by monotypy.
Tumbia Burn, 1962b: 161–163. Type species: Asteronotus (Tumbia) trenberthi Burn, 1962b [= Sclerodoristrenberthi (Burn, 1962b)], by monotypy.

##### Remarks.

For an in-depth discussion of the characteristics of the genus *Sclerodoris* and its synonyms see Valdés and Gosliner (2001).

#### ﻿ Sclerodoris tuberculata

Taxon classificationAnimaliaNudibranchiaDiscodorididae

Eliot, 1904

035E119B-8A75-57F0-85A8-5A99EBACFD40

**Figure 14.**
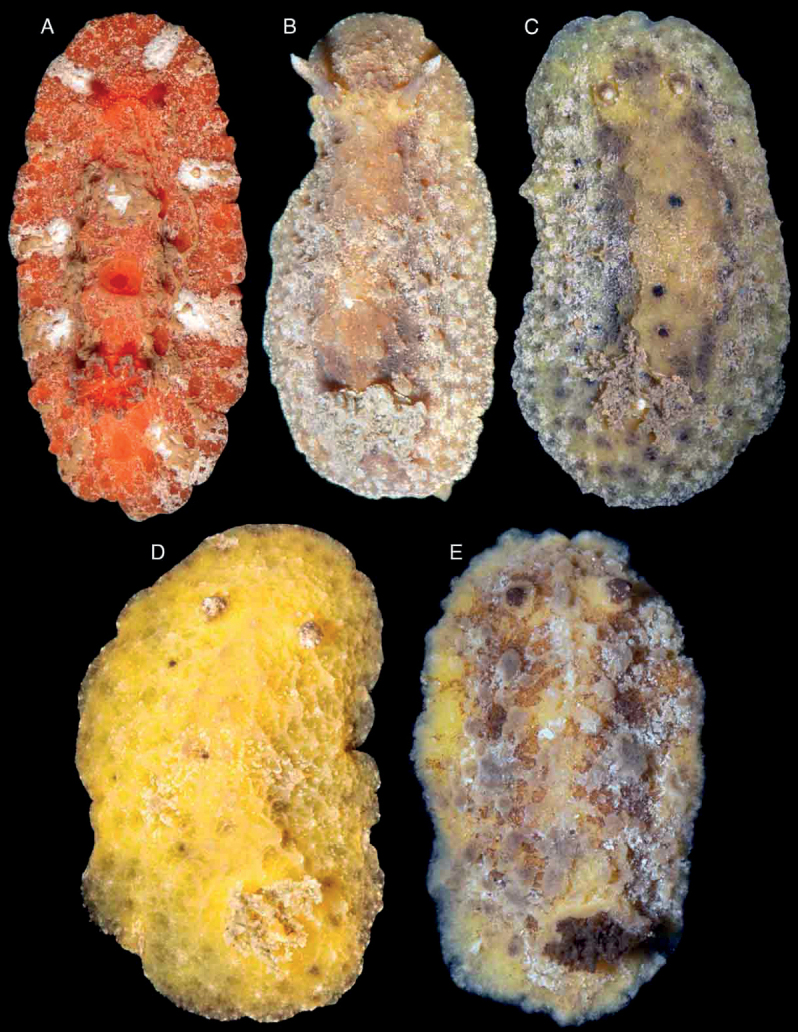
Photographs of live animals of the genus *Sclerodoris* Eliot, 1904 **A***Sclerodoristuberculata* Eliot, 1904, MNHN IM-2013-86197 on black background **B–D** “*Sclerodoris*” *dutertrei* sp. nov., Holotype (MNHN IM-2013-86193) on black background (**B**), MNHN IM-2013-86195 on black background (**C**), MNHN IM-2013-86194 on black background (**D**) **E***Sclerodorisfaninozi* sp. nov., Holotype (MNHN IM-2013-86198) on black background.

**Figure 15.**
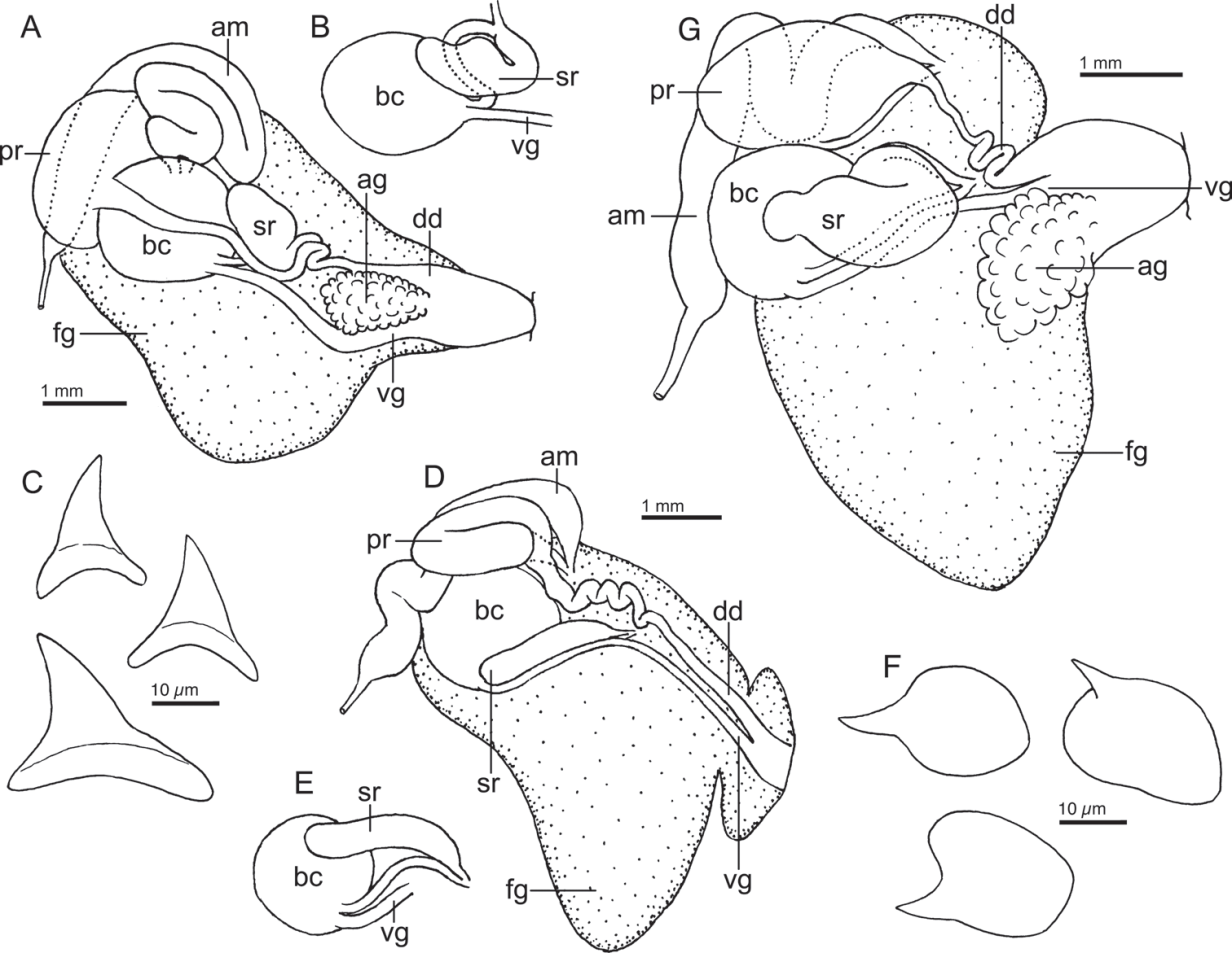
Drawing of the reproductive system of specimens of the genus *Sclerodoris* Eliot, 1904 **A–C***Sclerodoristuberculata* Eliot, 1904, MNHN IM-2013-86197, general view (**A**), detail of the bursa copulatrix and seminal receptable (**B**), penial spines (**C**) **D–F***Sclerodorisfaninozi* sp. nov., Holotype (MNHN IM-2013-86198), general view (**D**), detail of the bursa copulatrix and seminal receptable (**E**), penial spines (**F**) **G** “*Sclerodoris*” *dutertrei* sp. nov., MNHN IM-2013-86193. Abbreviations: ag, accessory gland; am, ampulla; bc, bursa copulatrix; dd, deferent duct; fg, female gland complex; pr, prostate; sr, seminal receptacle; vg, vagina.

**Figure 16.**
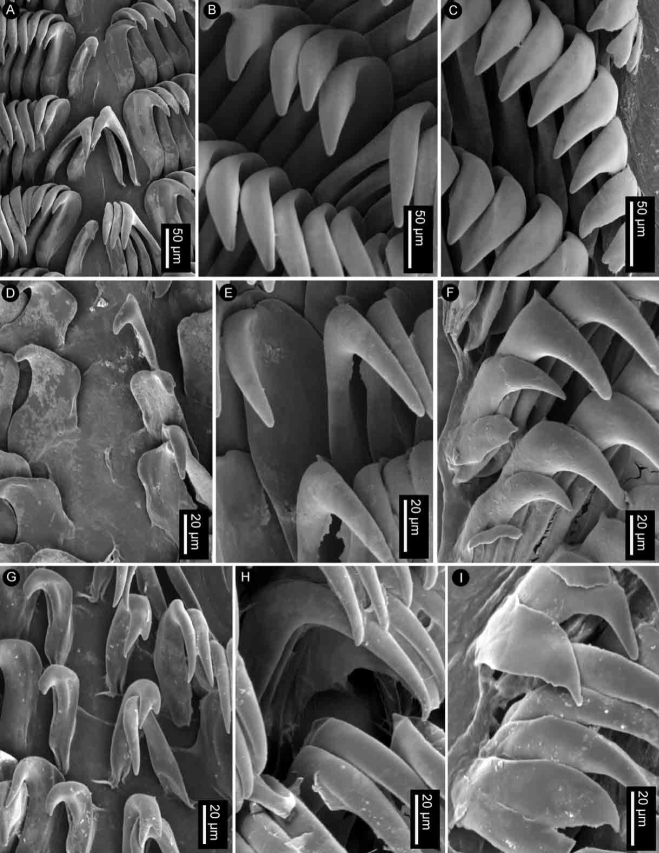
SEM of the radula of specimens of the genus *Sclerodoris* Eliot, 1904 **A–C***Sclerodoristuberculata* Eliot, 1904, MNHN IM-2013-86197, innermost teeth (**A**), mid-lateral teeth (**B**), outer lateral teeth (**C**) **D–F** “*Sclerodoris*” *dutertrei* sp. nov., MNHN IM-2013-86195, innermost teeth (**D**), mid-lateral teeth (**E**), outer lateral teeth (**F**) **G–I***Sclerodorisfaninozi* sp. nov., Holotype (MNHN IM-2013-86198), innermost teeth (**G**), mid-lateral teeth (**H**), outer lateral teeth (**I**).

Figs 14A , 15A–C , 16A–C

?Doriscastanea Kelaart, 1858: 110. Type locality: Sober Island, Tricomalie [= Trincomalee] harbor, Ceylon [= Sri Lanka].
Sclerodoris tuberculata Eliot, 1904: 381–382. Type locality: Prison Island [= Changuu], Zanzibar harbor, Tanzania.
Sclerodoris minor Eliot, 1904: 381. Type locality: Chuaka [= Chwaka], Zanzibar, Tanzania.
Sclerodoris rubra Eliot, 1904: 382–383. Type locality: reef off the east coast of Zanzibar, Tanzania.
Halgerda rubra Bergh, 1905: 126–127, pl. 4 fig. 2, pl. 15 figs 34–36. Type locality: Bandas [= Banda Islands], Indonesia, 36 m depth.

##### Material examined.

Pointe de Pandop, Koumac, New Caledonia (20°34.9'S, 164°16.5'E), 7 m depth [Koumac 2.1 stn. KR868], 26 Sep 2018, 1 specimen 44 mm long, dissected (MNHN IM-2013-86197, isolate JI10).

##### Description.

Body oval, flattened, with an irregular, coriaceous texture (Fig. 14A). Branchial and rhinophoral sheaths somewhat elevated, simple, circular. Gill composed of eight short, tripinnate branchial leaves, arranged upright. Rhinophores short, lamellated, with 18 lamellae. Visceral hump clearly elevated over the rest of the mantle, with several lateral protuberances and a conspicuous depression mid-length. Dorsum completely covered with small caryophyllidia. Body color red, with several large, irregularly opaque white patches, mainly on the mantle margin and some white pigment irregularly scattered all over. Rhinophores and branchial leaves are the same color as the dorsum.

Reproductive system (Fig. 15A, B) with a long, wide, convoluted ampulla with several folds, which connects with the female gland complex and the oval, flattened prostate. The prostate narrows substantially into a long, straight duct, before expanding into the short, wide deferent duct. The penis is armed with triangular spines, varying in size (Fig. 15C) with thickened bases and sharp cusps. The vagina is elongate, narrow, as wide as the deferent duct, connecting directly to the large, oval bursa copulatrix. The elongate seminal receptacle also connects to the bursa copulatrix next to the vaginal connection, and the short uterine duct that enters the female gland complex (Fig. 15B). The bursa copulatrix is ~ 4× as large as the seminal receptable. An accessory gland connects to the genial atrium where the deferent duct and the vagina meet. The accessory gland is granular in texture and approximately as large as the seminal receptable.

Radular formula 38 × 49.0.49 in a 44-mm long specimen (MNHN IM-2013-86197). Rachidian teeth absent. Inner and mid-lateral teeth hamate, having an elongate cusp and lacking denticles (Fig. 16A, B). Innermost teeth very small in comparison to mid-laterals (Fig. 16A). The teeth increase in size gradually towards the medial portion of the half-row. Outermost teeth small, decreasing in size gradually, composed of a short, blunt cusp with numerous small denticles (Fig. 13C). No jaw was observed, labial cuticle smooth.

##### Biology.

Rare, found under rocks at 7 m depth. Widespread in the Indo-Pacific region. The single specimen was found under a rock while SCUBA diving where it was highly cryptic.

##### Remarks.

Eliot (1904) described *Sclerodoristuberculata* based on one specimen collected in Zanzibar as follows: “Dark brown with sandy spots, exactly like a sponge splashed with sand. Underside clear bright brownish red. Branchial pocket crenulate. The middle part of back covered with conical warts, which form an irregular keel; smaller warts on mantle-edge. Rhinophores red; branchiae eight, voluminous; axes red, tips white. Animal alters shape, sometimes rather high, sometimes quite flat like *Platydoris*. Consistency quite hard and rather rough. Two depressions with deep black markings as in some species of *Trippa*.” In the same paper Eliot (1904) introduced two additional species also resembling sponges, *Sclerodorisminor* Eliot, 1904, and *Sclerodorisrubra* Eliot, 1904, both synonyms of *S.tuberculata*. *Sclerodoristuberculata* is considered a widespread species in the Indo-Pacific region and is well documented in the literature (Valdés and Gosliner 2001; Yonow 2008; Gosliner et al. 2018; Nakano 2018). The material here examined is consistent with the original description of *S.tuberculata* and subsequent records; however, a record of this species from New Caledonia (Hervé 2010) is probably the closely related species *Sclerodorisrubicunda* (Baba, 1949).

Eliot (1906) suggested that *Doriscastanea* Kelaart, 1858 was possibly the same species as *Sclerodoristuberculata* Eliot, 1904, but indicated the identity of the latter could not be established with certainty based on the type material. Eliot (1906: pl. 42, figs 6, 7) reproduced the original drawing by Kelaart, which clearly resembles a dark specimen of *S.tuberculata*. Later, Eliot (1908) regarded *Sclerodorisrubra* Eliot, 1904 as a senior synonym of *Halgerdarubra* Bergh, 1905.

Allan (1947) reported *S.tuberculata* from New South Wales, Australia, under the genus name *Peronodoris* Bergh, 1904 and commented on Eliot’s (1906) proposed synonymy between this species and *D.castanea*. Allan (1947) indicated that “although the colour sketch of the upper surface of Kelaart’s specimen resembles that of our specimen to a very slight degree,” the undersurface is exactly like the color sketch of the New South Wales material of *S.tuberculata*. Allan (1947) concluded that whether *S.tuberculata* was eventually to become a synonym of *D.castanea* remained to be seen, as fresh material from the two type localities needs to be examined before this can be determined.

Rudman (1978) endorsed Eliot’s (1908) decision to synonymize *Halgerdarubra* Bergh, 1905 with *Sclerodorisrubra* Eliot, 1904. At the same time Rudman (1978) regarded *Sclerodorisrubra* Eliot, 1904 and *Sclerodorisminor* Eliot, 1904 as synonyms of *Sclerodoristuberculata* Eliot, 1904, and based on the Principle of First Reviser (ICZN 1999: Article 24), Rudman (1978) established *S.tuberculata* as the valid name for this species. Rudman (1978) also commented that the original description of *D.castanea* by Kelaart (1858) was most inadequate and therefore best to ignore it. In this paper we follow Rudman’s (1978) conclusion and regard *Sclerodoristuberculata* Eliot, 1904 as the valid name for this species with the synonymies established above. We also leave the question of the identity of *D.castanea* as unresolved.

Hervé (2010) reported *Sclerodoristuberculata* from New Caledonia but based on the photographs published (Hervé 2010: 214), it seems that these records correspond to *Sclerodorisrubicunda* (Baba, 1949). The present study is the first confirmed record of *Sclerodoristuberculata* from New Caledonia.

#### ﻿ Sclerodoris faninozi sp. nov.

Taxon classificationAnimaliaNudibranchiaDiscodorididae

766038AF-CEBA-5A5E-BBF0-D528D13C26D5

https://zoobank.org/619B72BC-611E-4E53-ABAA-0D8D67284448

Figs 14E , 15D–F , 16G–I

##### Type material.

***Holotype***: Koumac, New Caledonia (20°33.7'S, 164°11.2'E), 0 m depth [Koumac 2.3 stn. KB518, blocks of dead coral on the margin of the fringing reef flat of the lagoon island], 20 Nov 2019, 25 mm long, dissected (MNHN IM-2013-86198, isolate JI11).

##### Description.

Body oval, flattened, with an irregular, coriaceous texture (Fig. 14E). Branchial and rhinophoral sheaths somewhat elevated, simple, irregular. Gill composed of five short, tripinnate branchial leaves, arranged upright. Rhinophores short, lamellated, with 15 lamellae. Visceral hump elevated over the rest of the mantle. Dorsum completely covered with small caryophyllidia, a longitudinal ridge, and several large, rounded tubercles. Body color yellowish brown, with scattered opaque white pigment, and areas or dark brown and dark gray. Branchial leaves and rhinophores dark brown.

Reproductive system (Fig. 15D, E) with a long, wide, convoluted ampulla with several folds, which connects with the female gland complex and the elongate, convoluted prostate. The prostate is as wide as the ampulla, but narrows substantially into a very long duct, before expanding into the long, narrow deferent duct. The penis is armed with rounded spines having a short, sharp cusp (Fig. 15F). The vagina is elongate, narrow, as wide as the deferent duct, connecting directly to the large, spherical bursa copulatrix. The elongate seminal receptacle also connects to the bursa copulatrix and the uterine duct that enters the female gland complex. The bursa copulatrix is ~ 3× wider than the seminar receptable, but similar in volume (Fig. 15D). No accessory gland was observed.

Radular formula 32 × 68.0.68 in a 25-mm long specimen (MNHN IM-2013-86198). Rachidian teeth absent. Inner and mid-lateral teeth hamate, having an elongate cusp (sometimes bifurcate) and lacking denticles (Fig. 16G–I). Innermost teeth very small in comparison to mid-laterals (Fig. 16G). The teeth increase in size gradually towards the medial portion of the half-row. Outermost teeth small, decreasing in size gradually, elongate, with a short cusp and numerous denticles (Fig. 13I). No jaw was observed, labial cuticle smooth.

##### Biology.

Rare, found intertidally under rocks, possibly a New Caledonia endemic. The single specimen was obtained by brushing blocks of dead coral on the margin of a fringing reef flat.

##### Etymology.

This species is named after Sébastien Faninoz whose efforts were critical for the organization of the Koumac expeditions.

##### Remarks.

In the phylogenetic analyses conducted herein, *Sclerodorisfaninozi* sp. nov. is sister to *Sclerodoristuberculata*, the type species of *Sclerodoris*, forming a well-supported clade; for this reason, *S.faninozi* sp. nov. is placed in the genus *Sclerodoris*. Moreover, most of the anatomical characteristics of *S.faninozi* sp. nov. match the diagnosis of the genus *Sclerodoris* provided by Valdés and Gosliner (2001). Specifically, *S.faninozi* sp. nov. has a flattened, coriaceous dorsum covered with caryophyllidia, the rhinophoral sheaths are somewhat elevated; the penis is armed with hooks and the vagina is unarmed; the labial cuticle and radular teeth are smooth, hamate with the outermost lateral teeth multi-denticulate. The only exception is the accessory gland, which is a diagnostic trait for *Sclerodoris*, but was not observed in *S.faninozi* sp. nov. Although the absence of an accessory gland in *S.faninozi* sp. nov. could have been result of damage to the specimen, it appears that the presence of this organ is variable in *Sclerodoris*.

*Sclerodorisfaninozi* sp. nov. is externally similar to *Sclerodoriscoriacea* Eliot, 1904 introduced based on a specimen collected near Chwaka (as Chuaka), on the east coast of Zanzibar, Tanzania. Eliot (1904) described *S.coriacea* as yellowish brown in color with the dorsal surface covered with a “distinctly raised but somewhat irregular reticulate pattern.” Rudman (1978) redescribed *S.coriacea* also based on specimens from Zanzibar, and a color photograph of a live animal was illustrated by Gosliner et al. (2018). The specimen of *S.faninozi* sp. nov. here examined is similar to all these descriptions with the exception of the presence of a dorsal ridge, absent in *S.coriacea*. The radular morphology of *S.faninozi* sp. nov. is also similar to that of *S.coriacea* as described by Rudman (1978) but the innermost teeth of *S.faninozi* sp. nov. have a bifurcated cusp, whereas they are simple in *S.coriacea* (Rudman 1978: fig. 13).

#### ﻿“ Sclerodoris dutertrei

Taxon classificationAnimaliaNudibranchiaDiscodorididae

” sp. nov.

84A08E53-66E8-5799-9D7A-A59DEDE231D9

https://zoobank.org/504AD504-89AE-48D4-9840-4800830CC0AC

Figs 14B–D , 15G , 16D–F

##### Type material.

***Holotype***: Anse de Koumac, New Caledonia (20°34.2'S, 164°16.5'E), 0 m depth [Koumac 2.1 stn. KR213], 11 Sep 2018, 31 mm long (MNHN IM-2013-86193, isolate JI04).

##### Material examined.

Récif Sud de Pandop, Koumac, New Caledonia (20°35.4'S, 164°16.5'E), 0 m depth [Koumac 2.1 stn. KR322, reef flat with rocks, living and dead corals], 27 Sep 2018, 1 specimen 23 mm long (MNHN IM-2013-86196, isolate JI14). Koumac, New Caledonia (20°35.6'S, 164°16.3'E), 3 m depth [Koumac 2.2 stn. KR230], 2 Mar 2019, 1 specimen 12 mm long (MNHN IM-2013-86194, isolate JI35); 1 specimen 20 mm long, dissected (MNHN IM-2013-86195, isolate JI34).

##### Description.

Body oval, flattened, with an irregular, coriaceous texture (Fig. 14B–D). Branchial and rhinophoral sheaths somewhat elevated, simple, irregular. Gill composed of five short, tripinnate branchial leaves, arranged upright. Rhinophores short, lamellated, with 12–14 lamellae. Visceral hump elevated over the rest of the mantle. Dorsum completely covered with small caryophyllidia and a complex network of ridges and scattered large, rounded tubercles. Body color variable, yellow to pale brown with scattered opaque white pigment and some specimens with rounded black spots. Branchial leaves are the same color as the dorsum; rhinophores brown proximally, with white apices.

Reproductive system (Fig. 15G) with a long, wide, convoluted ampulla with several folds, which connects with the female gland complex and the oval, flattened prostate. The prostate narrows substantially into a long, convoluted duct, before expanding into the short, wide deferent duct. The penis is unarmed. The vagina is elongate, much narrower than the deferent duct, connecting directly to the large, oval bursa copulatrix. The elongate seminal receptacle also connects to the bursa copulatrix next to the vaginal connection, and the short uterine duct that enters the female gland complex. The seminal receptable possesses a spherical tip and it is similar in volume to the bursa copulatrix. An accessory gland connects to the genial atrium where the deferent duct and the vagina meet. The accessory gland is granular in texture and approximately as large as the bursa copulatrix.

Radular formula 37 × 54.0.54 in a 20-mm long specimen (MNHN IM-2013-86195). Rachidian teeth absent. Inner and mid-lateral teeth hamate, having a short cusp and lacking denticles (Fig. 16D, E). Innermost teeth very small in comparison to mid-laterals (Fig. 16D). The teeth increase in size gradually towards the medial portion of the half-row. Outermost teeth small, decreasing in size gradually, elongate, with a short cusp and lacking differentiated denticles (Fig. 16F). No jaw was observed, labial cuticle smooth.

##### Biology.

Found under rocks at 0–3 m depth. All the specimens were obtained by direct collection while SCUBA diving. The specimens were very cryptic on rocks with sponges and other encrusting organisms.

##### Etymology.

This species is named after Valentine Dutertre whose hard work, dedication, and skill were critical for the collection of numerous important sea slug species during the Koumac expeditions.

##### Remarks.

The phylogenetic analysis places “*Sclerodoris*” *dutertrei* sp. nov. in a well-supported clade containing two other species identified as members of *Sclerodoris*. These two species were sequenced and submitted to GenBank but never formally studied, thus their morphological characteristics remain undescribed. This clade is not closely related to the clade containing the rest of the species of *Sclerodoris*, including the type species, *Sclerodoristuberculata*. Therefore, “*S.*” *dutertrei* sp. nov. cannot be definitely included in the genus *Sclerodoris* and the generic placement of this species is regarded as tentative until a well resolved phylogeny of the Discodorididae permits a more accurate taxonomic placement. “*Sclerodoris*” *dutertrei* sp. nov. is tentatively placed in *Sclerodoris* (as indicated by the quotation marks) because anatomically this species is for the most part consistent with the diagnosis for *Sclerodoris* provided by Valdés and Gosliner (2001), including a flattened, coriaceous dorsum covered with caryophyllidia, rhinophoral sheaths somewhat elevated; a lobate accessory gland, without stylet; labial cuticle and radular teeth smooth, hamate with the outermost lateral teeth multidenticulate. The only exception is the penis, which appears to be unarmed in “*S.*” *dutertrei* sp. nov., but the presence of penial spines is a characteristic of *Sclerodoris* sensu stricto (see Valdés and Gosliner 2001).

“*Sclerodoris*” *dutertrei* sp. nov. is distinct from other species previously assigned to *Sclerodoris*: no other species described to date possesses a yellow to pale brown dorsum with scattered opaque white pigment (sometimes with rounded black spots), completely covered with small caryophyllidia and a complex network of ridges and scattered large, rounded tubercles. As mentioned above, *Sclerodoristuberculata* is red with several large, irregularly shaped, opaque white patches and a conspicuous depression mid-length on the dorsum, not present in “*Sclerodoris*” *dutertrei* sp. nov.; *Sclerodorisfaninozi* sp. nov. is yellowish brown, with scattered opaque white pigment, and areas of dark brown and dark gray but also has a longitudinal ridge, and several large, rounded tubercles, also absent in “*Sclerodoris*” *dutertrei* sp. nov. Other Indo-Pacific species described also present external characteristics that distinguish them from “*Sclerodoris*” *dutertrei* sp. nov. For example, *Sclerodorisapiculata* (Alder & Hancock, 1864) is characterized by having a network of ridges radiating from elevated conical centers, each with an elongated filament (see Alder and Hancock 1864; Hervé 2010; Gosliner et al. 2018; Nakano 2018). *Sclerodoriscoriacea* has the dorsum completely covered with large, elongate tubercles joined by conspicuous ridges (see Rudman 1978; Gosliner et al. 2018), very different from those in “*Sclerodoris*” *dutertrei* sp. nov. *Sclerodorisjaponica* (Eliot, 1913), originally described as a member of the genus *Halgerda* (see Eliot 1913) is characterized by having a yellowish grey dorsum covered with small ridges, and numerous, large roundish areas of a darker grey, varying in intensity, which correspond to dorsal depressions or pits. *Sclerodorisrubicunda* is a red species with two large patches of white and purple pigment and a series of conspicuous dorsal ridges (Baba 1949; Gosliner et al. 2018; Nakano 2018). *Sclerodoristrenberthi* (Burn, 1962b) and *Sclerodoristarka* Burn, 1969 both described from Victoria, Australia are also distinct from “*Sclerodoris*” *dutertrei* sp. nov. *Sclerodoristrenberthi* has a characteristic longitudinal dorsal structure composed of “irregularly sized and spaced low hard pustules surmounting a low ridge” running from the rhinophores to the gill (Burn 1962b), which is absent from “*Sclerodoris*” *dutertrei* sp. nov. *Sclerodoristarka* is a dusky yellow to yellowish orange species with a pattern of conspicuous dorsal ridges (Burn, 1969) and an indistinct medial ridge, also absent in “*Sclerodoris*” *dutertrei* sp. nov. Finally, *Sclerodorisvirgulata* Valdés, 2001 is the only species of *Sclerodoris* with a white dorsum lacking dorsal ridges or depressions (Valdés 2001), also very different from “*Sclerodoris*” *dutertrei* sp. nov.

## ﻿Discussion

The phylogeny presented here is largely consistent with previous morphological studies and the classification of the Discodorididae proposed by Valdés and Gosliner (2001) and Valdés (2002) with some exceptions. For example, the genus *Atagema* is sister to the rest of Discodorididae + Cadlinidae, but due to the poor representation of Cadlinidae in this study, these results should be taken cautiously. There is also a discrepancy with the molecular analysis by Hallas et al. (2017), who found *Atagema* + *Aphelodoris* as sister to remaining members of Discodorididae, but *Aldisa* + *Cadlina* forming a distinct clade, as also recovered by Johnson (2010) and Johnson and Gosliner (2012). On the contrary, in the present analyses *Aldisa* is nested within the Discodorididae. The more limited taxon sampling in the present study could explain this discrepancy, but the goal of the present analysis is only to place the new species here described in a phylogenetic context, not to provide a reliable reconstruction of the phylogeny of Discodorididae, which may only be achieved with next generation sequence data. There are some other differences between the present analyses and previous classification attempts of species included herein. For example, *Discodoriscoerulescens* was regarded by Dayrat (2010) as a member of a metaphyletic group branching from near the basal node of Discodorididae he named “*Montereina*,” but the present analyses appear to suggest a close relationship with the genus *Tayuva* Er. Marcus and Ev. Marcus, 1967. *Tayuva* was considered a synonym of *Discodoris* by Valdés (2002) and *T.lilacina*, originally described as *Dorislilacina* Gould, 1852, is regarded as a member *Discodoris* by some authors (e.g., Gosliner et al. 2018); however, other authors following Dayrat (2010) placed this species in *Tayuva*, a distinct genus with a single pantropical species (e.g., Ballesteros et al. 2016; Yonow 2017). The results of the present analysis appear to confirm that *Tayuva* is distinct from *Discodoris* as suggested by Dayrat (2010), but it is unclear how many species are present in this pantropical complex. Finally, the genus *Montereina* MacFarland, 1905 was synonymized with *Peltodoris* by Valdés (2002), but the results of the present analyses suggest that these two groups are distinct as suggested by Dayrat (2010).

Based on the phylogenetic analyses here presented, it appears that the genus *Sclerodoris* is paraphyletic. The new species “*Sclerodoris*” *dutertrei* sp. nov. was recovered in a well-supported clade containing two other species identified as members of *Sclerodoris*, but not in the clade including *Sclerodoristuberculata* Eliot, 1904, which is the type species of *Sclerodoris*. Thus, the description of a new genus name for the clade including “*Sclerodoris*” *dutertrei* sp. nov. is an option. However, due to the limited sample size in our molecular phylogenies and the lack of support for several clades, we prefer to postpone any decisions regarding this group until a more reliable phylogeny of the Discodorididae is available, as there could be available genus-level names for this group. Therefore, the generic placement of “*Sclerodoris*” *dutertrei* sp. nov. is regarded as tentative, indicated by the quotation marks.

Bouchet et al. (2007) argued that “it can safely be affirmed that, as a result of the recent sampling programs, both in shallow and in deep-water, no other South Pacific island group has been so intensively surveyed as New Caledonia.” However, recent field work during the Koumac expeditions seems to have revealed additional diversity missed during early work, suggesting that documenting the New Caledonia molluscan diversity is still a work in progress. As Bouchet et al. (2007) indicated, the question of how many mollusk species are present in New Caledonia remains unanswered and this is particularly true for sea slugs. This paper is a small contribution towards the goal of describing the sea slug diversity of New Caledonia as field work continues to produce previously unseen taxa.

It is unclear how many of the species here described are endemic to New Caledonia. Payri et al. (2019) suggested that probably < 15% of the New Caledonia marine mollusks are endemic, although they also indicated that “several scientists have already demonstrated connections between the marine life of New Caledonia, the Great Barrier reef, and the center of maximum diversity of the Coral Triangle.” Based on photographs published in field guides or other publications, it is likely that *Atagemapapillosa* (Risbec, 1928), *Atagemasobanovae* sp. nov., and *Jorunnadaoulasi* sp. nov. are widespread in the Western Pacific, but we have been unable to find photographs of *Atagemakimberlyae* sp. nov., *Jorunnahervei* sp. nov., *Rostangapoddubetskaiae* sp. nov., *Sclerodorisfaninozi* sp. nov., and “*Sclerodoris*” *dutertrei* sp. nov. in other publications outside New Caledonia. But due to the very cryptic nature of these species, it could very well be that they have been overlooked. While the small size of the eggs of *J.hervei* sp. nov. suggests planktotrophic development and therefore a potentially large geographic range, the recent description of a very similar species from the Indian Ocean, *J.liviae*, may indicate there is a species complex of species with similar external morphologies present in different ocean basins. Much more work on these neglected dorid nudibranchs is needed to have a better understanding of their taxonomy, diversity, and evolution.

The specimens here examined where collected using different techniques, including dredging, direct collecting (intertidally and SCUBA diving), and substrate collecting. Due to the highly cryptic coloration and morphology of some of the species, their presence was detected initially by the observation of egg masses on the sponges. In the particular case of *A.sobanovae* sp. nov., most of the specimens were collected by dissecting the sponges in the laboratory as the nudibranchs were buried in the tissue, and almost invisible. The diversity of collecting techniques and specialized methods used during the Koumac expeditions were critical in the discovery of the species here examined. This paper provides a rare example of the description and re-description of ecologically cryptic sea slug species using contemporary taxonomic techniques and focusing on a narrow geographic region that, despite substantial collecting efforts (Bouchet et al. 2007), appears to remain under-sampled.

## Supplementary Material

XML Treatment for Atagema

XML Treatment for Atagema spongiosa

XML Treatment for Atagema papillosa

XML Treatment for Atagema kimberlyae

XML Treatment for Atagema sobanovae

XML Treatment for Jorunna

XML Treatment for Jorunna daoulasi

XML Treatment for Jorunna hervei

XML Treatment for Rostanga

XML Treatment for Rostanga poddubetskaiae

XML Treatment for Sclerodoris

XML Treatment for Sclerodoris tuberculata

XML Treatment for Sclerodoris faninozi

XML Treatment for ﻿“ Sclerodoris dutertrei

## Supplementary materials

Supplementary material 1 Individual analysis of COI gene fragments Data type figure (jpg file) Explanation note Posterior probabilities are shown above the branches and bootstrap values from the maximum-likelihood analysis values shown below branches. https://binary.pensoft.net/file/820845This dataset is made available under the Open Database License (http://opendatacommons.org/licenses/odbl/1.0/). The Open Database License (ODbL) is a license agreement intended to allow users to freely share, modify, and use this Dataset while maintaining this same freedom for others, provided that the original source and author(s) are credited.Julie Innabi, Carla C. Stout, Ángel Valdés

Supplementary material 2 Individual analysis of 16S gene fragments Data type figure (jpg file) Explanation note Posterior probabilities are shown above the branches and bootstrap values from the maximum-likelihood analysis values shown below branches. https://binary.pensoft.net/file/820846This dataset is made available under the Open Database License (http://opendatacommons.org/licenses/odbl/1.0/). The Open Database License (ODbL) is a license agreement intended to allow users to freely share, modify, and use this Dataset while maintaining this same freedom for others, provided that the original source and author(s) are credited.Julie Innabi, Carla C. Stout, Ángel Valdés

Supplementary material 3 Individual analysis of Histone H3 gene fragments Data type figure (jpg file) Explanation note Posterior probabilities are shown above the branches and bootstrap values from the maximum-likelihood analysis values shown below branches. https://binary.pensoft.net/file/820847This dataset is made available under the Open Database License (http://opendatacommons.org/licenses/odbl/1.0/). The Open Database License (ODbL) is a license agreement intended to allow users to freely share, modify, and use this Dataset while maintaining this same freedom for others, provided that the original source and author(s) are credited.Julie Innabi, Carla C. Stout, Ángel Valdés
