# Supplementary figures and images for: ﻿Seven new “cryptic” species of Discodorididae (Mollusca, Gastropoda, Nudibranchia) from New Caledonia
Source: Zookeys. 2023 Mar 7;1152:45–95. doi: 10.3897/zookeys.1152.98258 (PMC10194415; doi:10.3897/zookeys.1152.98258)

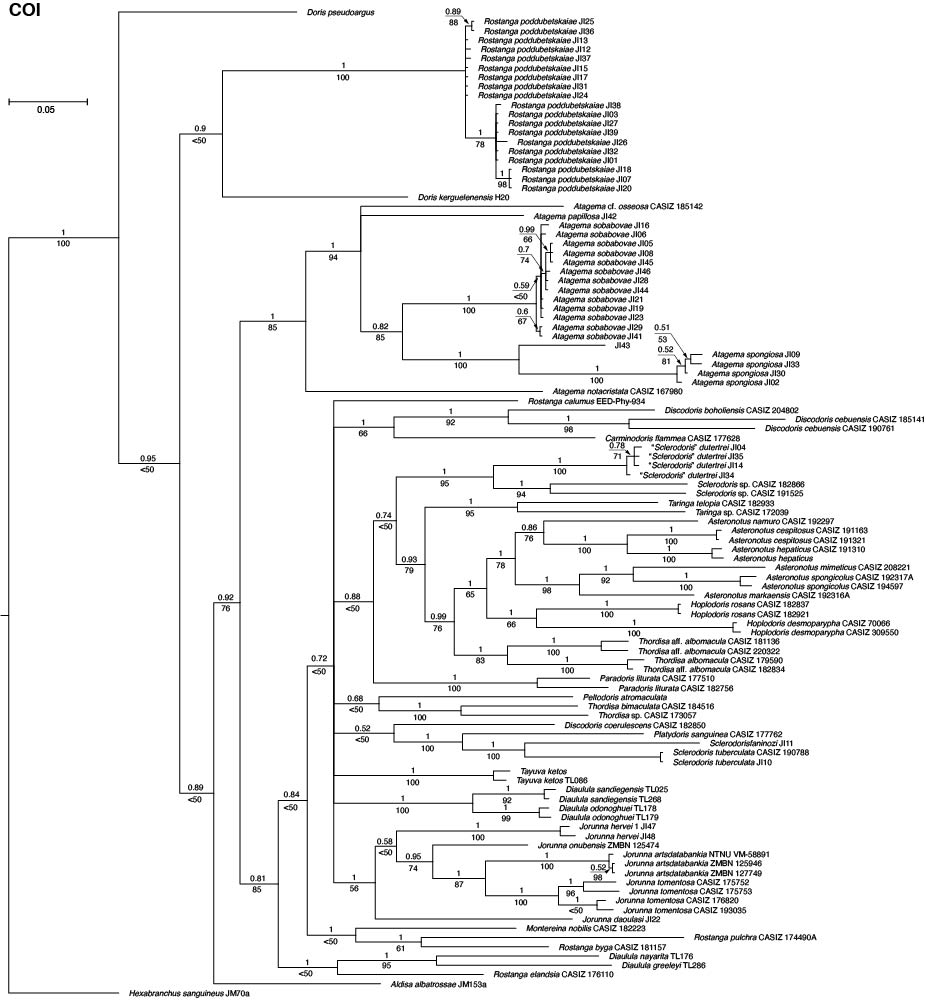

Supplement: Supplementary material 1 — Individual analysis of COI gene fragments [file zookeys-1152-045_article-98258__-s001.jpg]

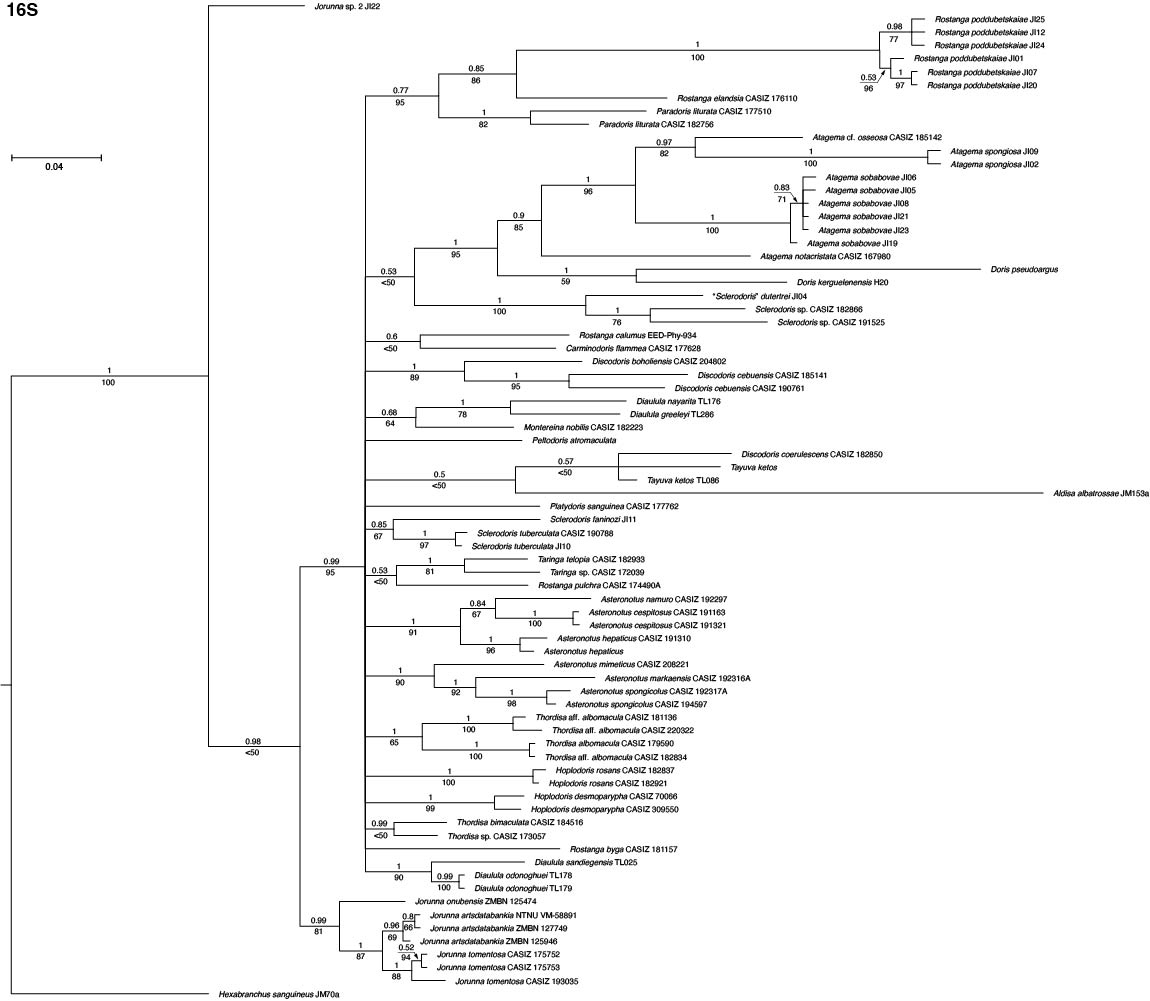

Supplement: Supplementary material 2 — Individual analysis of 16S gene fragments [file zookeys-1152-045_article-98258__-s002.jpg]

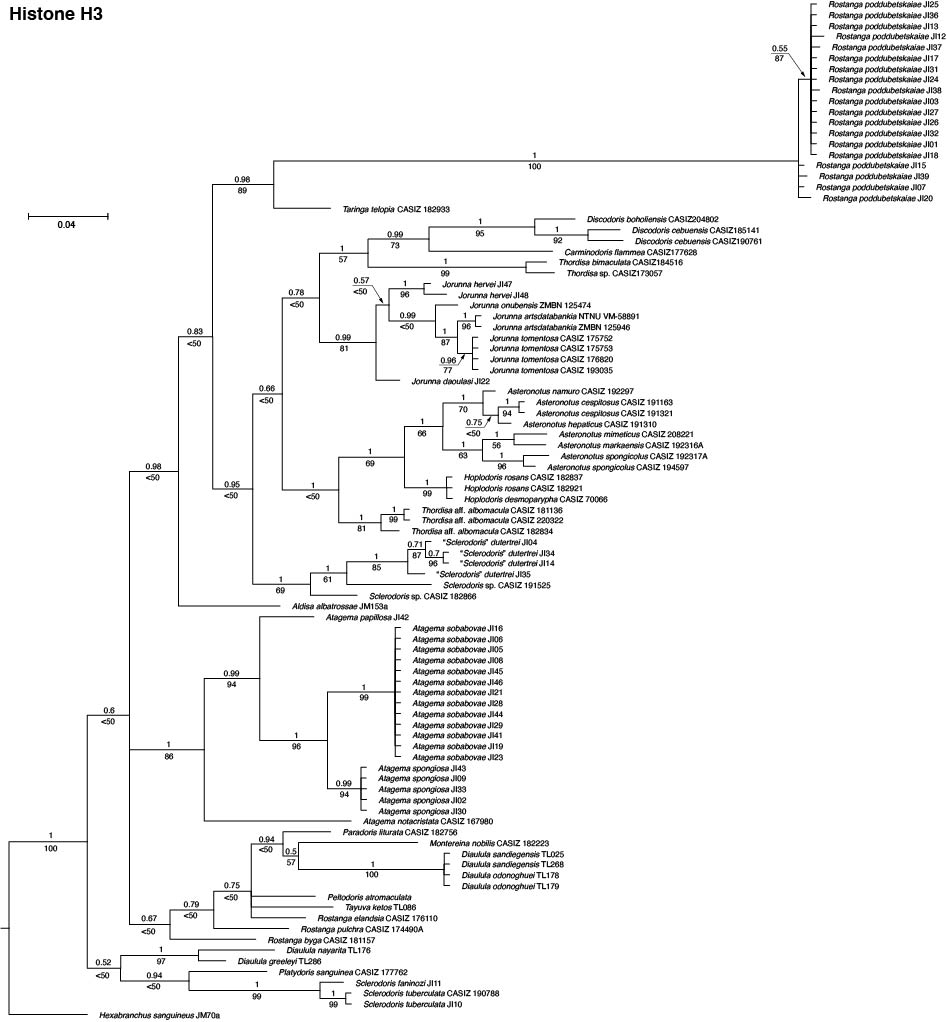

Supplement: Supplementary material 3 — Individual analysis of Histone H3 gene fragments [file zookeys-1152-045_article-98258__-s003.jpg]
